# Targeted knock-in mice expressing the oxidase-fixed form of xanthine oxidoreductase favor tumor growth

**DOI:** 10.1038/s41467-019-12565-z

**Published:** 2019-10-28

**Authors:** Teruo Kusano, Driss Ehirchiou, Tomohiro Matsumura, Veronique Chobaz, Sonia Nasi, Mariela Castelblanco, Alexander So, Christine Lavanchy, Hans Acha-Orbea, Takeshi Nishino, Ken Okamoto, Nathalie Busso

**Affiliations:** 1 Department of Biochemistry and Molecular Biology, Nippon Medical School, 1-1-5, Sendagi, Bunkyo-Ku, Tokyo Japan; 2 Department of Biochemistry CIIL, University of Lausanne, Epalinges, Switzerland; 3 DAL, Service of Rheumatology, Laboratory of Rheumatology, University of Lausanne, CHUV, Epalinges, Switzerland; 4 Present Address: Isotope Research Laboratory, Nippon Medical School, 1-1-5 Sendagi, Bunkyo-ku, Tokyo Japan; 5 Present Address: Department of Applied Biological Chemistry, Graduate School of Agricultural and Life Sciences, The University of Tokyo, 1-1-1 Yayoi, Bunkyo-ku, Tokyo Japan

**Keywords:** Cancer, Tumour immunology

## Abstract

Xanthine oxidoreductase has been implicated in cancer. Nonetheless, the role played by its two convertible forms, xanthine dehydrogenase (XDH) and oxidase (XO) during tumorigenesis is not understood. Here we produce XDH-stable and XO-locked knock-in (ki) mice to address this question. After tumor transfer, XO ki mice show strongly increased tumor growth compared to wild type (WT) and XDH ki mice. Hematopoietic XO expression is responsible for this effect. After macrophage depletion, tumor growth is reduced. Adoptive transfer of XO-ki macrophages in WT mice increases tumor growth. In vitro, XO ki macrophages produce higher levels of reactive oxygen species (ROS) responsible for the increased Tregs observed in the tumors. Blocking ROS in vivo slows down tumor growth. Collectively, these results indicate that the balance of XO/XDH plays an important role in immune surveillance of tumor development. Strategies that inhibit the XO form specifically may be valuable in controlling cancer growth.

## Abstract (web summary)

The roles of the convertible forms, xanthine dehydrogenase (XDH) and xanthine oxidase (XO) during tumorigenesis is not known. Here, the authors develop XDH-stable and XO-locked knock-in (ki) mice and show increased tumor growth in XO ki mice, via macrophage-mediated immunoregulatory responses.

## Introduction

XOR is an enzyme that is expressed in various organs including liver, kidney, intestine, mammary glands, and capillary endotherial cells. The enzyme is a homodimeric protein with a relative molecular mass of 290,000 and is composed of independent subunits; each subunit containing one molybdopterin (Moco center), two nonidentical [2Fe-2S] type iron sulfur clusters (Fe/S centers) and one FAD as cofactors^1,2^. The enzyme catalyzes the hydroxylation of hypoxanthine to xanthine, and xanthine to uric acid at the Moco center, and two electrons are transferred to the Mo atom (Mo^VI^ to Mo^IV^) of Moco in each reaction, thus introduced that the electrons are further transferred rapidly to FAD via two Fe/S centers, and reduction of NAD^+^ or oxygen occurs^2–4^. Mammalian XOR can be found in two inter-convertible forms: xanthine dehydrogenase (XDH, EC 1.1.1.204), which is the genetically coded form, which uses NAD^+^ as a cofactor, and xanthine oxidase (XO, EC 1.1.3.22), which uses oxygen. Whereas XDH activity leads to NADH production, XO activity generates ROS, such as superoxide anions (O_2_^−^) and hydrogen peroxide, which are involved in diverse physiological roles, e.g., in host defense against infectious pathogens, or in the pathology of postischemic reperfusion injury^5–7^. In addition, results from XOR-deficient mice demonstrated an important physiological role of the XO form in lactation^8^. Since then, additional physiological and pathological roles of XOR-derived ROS have been found^2,9^.

The conversion between XDH and XO can occur either irreversibly by limited proteolysis, or reversibly by oxidation of XDH thiol groups^10–12^, and structural differences and mechanisms of conversion between the two forms were assessed by mutagenesis studies and crystal structural studies^10–17^. It has been shown that the conversion of the XDH form to the XO form leads to cytotoxicity and death of the damaged cells^18^. Accordingly, XOR inhibitors, such as febuxostat and allopurinol, have shown a protective effect in diseases such as atherosclerosis and chronic heart failure^19^.

XOR has also been implicated both in the pathogenesis^20^ and the prevention^21^ of cancer through the action of XOR-derived ROS^22^. A decrease of XOR activity has been found in tumor cells leading to a shift in the purine anabolic-catabolic balance, which should confer selective advantages to malignant cells.^23^. XOR down-modulation has been linked to more aggressive breast cancer^24^ and pharmacologic inhibition of XOR increased tumor burden in a mouse xenograft model^25^. Furthermore, XO and also XDH under hypoxic conditions are a source of ROS and subsequent DNA damage, which can cause malignant tumors, reported in several studies^26,27^. Although the involvement of XOR in cancer has been proposed, the mechanisms that link the XDH and XO forms to tumor-related inflammatory pathways are not clear.

The physiological and pathological roles of the XDH/XO conversion remain controversial. One key reason is the conversion of XDH to XO by lysosomal protease or oxidation of sulfhydryl groups during sample preparation, making the evaluation of when, where, and how much of XOR exists in the XO form very difficult to assess. Here, we constructed XOR gene-modified mice to examine the function of the two XOR forms. Based on previous mutagenesis studies and X-ray structural determination of rat enzyme^28,29^, we constructed stable XDH type mutant (C995R) and XO-locked type mutant (W338A/F339L) knock-in mice. To address the specific contribution of XDH and XO in cancer, we have investigated tumor growth after syngenic tumoral cells transfer in XDH ki and XO ki mice. In XO ki mice tumor growth was strikingly enhanced, which was dependent on XO ki macrophages and could be prevented by blocking the excessive ROS produced by XO ki macrophages. As a result, increased numbers of tumor infiltrating Tregs were found responsible for enhanced tumor growth.

## Results

### Generation of XOR mutant knock-in mice

**Fig. 1:**
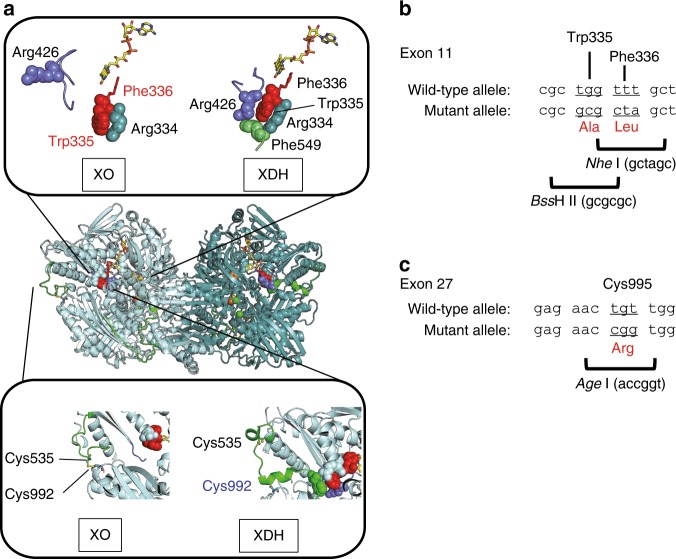
Design and construction of mouse XO ki and XDH ki mutants. **a** Mutant structures are designed from rat XOR W335A and F336L double mutant (PDB ID: 2E3T), and rat XOR C535A, C992R, and C1324S triple mutant (PDB ID: 1WYG). Amino acid cluster consisted of R334, W335, R426, and F549 are shown in space fill model. Upper inset, Active site loop (Gln422-Lys432) is shown in light blue. Corresponding residues to those mutated in XO ki mice are shown in red. Lower inset, Crystal structure around Cys535 in the loop connecting FAD and Molybdenum domains (green color). Cys992 in the molybdenum domain corresponding to the mutated residue in XDH ki mice is shown in cyan. **b** Targeted mutation sites of the murine Xdh gene for XO ki. The W338A/F339L mutation was introduced into exon 11. Minor differences in numbering of amino acids in mice used in this study are due to minor changes of amino acid sequences between rat and mouse. Therefore, W338 and F339 residues of murine XOR correspond to W335 and F336 residues of rat enzyme, respectively. **c** Targeted mutation sites of the murine Xdh gene for XDH ki. The C995R mutation introduced into exon 27. C995 residue of murine XOR corresponds to C992 residue of the rat enzyme

**Fig. 2:**
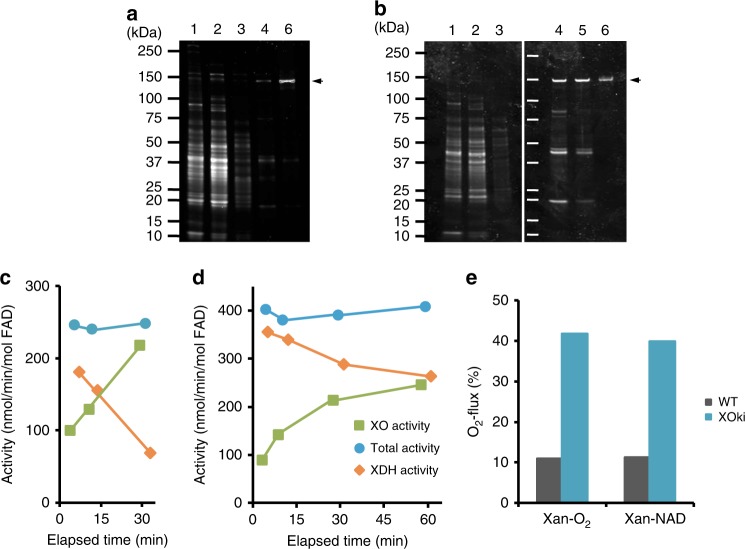
Verification of the expression in the XOR mutant ki mice. Details of mouse liver XOR purification were described in the “Methods” section. **a** SDS-PAGE analysis of each step of XOR purification from XO ki mouse liver; **b** SDS-PAGE analysis of each step of XOR purification from XDH ki mouse liver. Analysis was performed in a 5–20% polyacrylamide gel. Lane 1, liver cytosol fraction; lane 2, ammonium sulfate fractionation (20–55%); lane 3, anion exchange column (DE 52) fraction; lane 4, calcium phosphate column (Macro-Prep ceramic hydroxyapatite) fraction; lane 5, folate-affinity column side-fraction. Lane 6, folate-affinity column fraction. Lanes 1, 2, and 3 contain 2 µg of protein. Lanes 4, 5, and 6 contain 200 ng of protein. Protein bands in the electrophoresis gel were stained with Oriole. The arrowhead on the right side indicates the protein band derived from XOR. The molecular masses of the size standards are marked on the left side in kilodaltons. Purified XORs from the mutant mice were characterized to verify the proper expression of mutant XOR enzymes. To identify the XDH-stable property, purified XOR from XDH ki mice was analyzed. **c** Conversion of bovine milk native-XDH to XO by chemical modification. **d** Conversion from XDH to XO of XDH ki XOR by chemical modification. 4,4′-Dithiodipyridine was reacted with XDH form enzyme in 50 mM sodium phosphate buffer, pH 7.4 at 25 °C. Reactants were withdrawn after incubation at indicated intervals, and O_2_-dependent urate formation, NAD^+^-dependent urate formation, and NAD^+^-dependent NADH formation activities were assayed. Detail of assays was as described in the “Methods” section. **e** Comparision of O_2_^−^ production ratio during XOR turnover. The XO form of the purified mouse XOR enzyme was used in the assay. The activity of cytochrome c reduction was a difference between the presence and absence of superoxide dismutase, and the value indicated O_2_^−^ formation activity. O_2_^−^ flux is the percentage at which electrons generated by oxidation of xanthine flowed into O_2_^−^

**Fig. 3:**
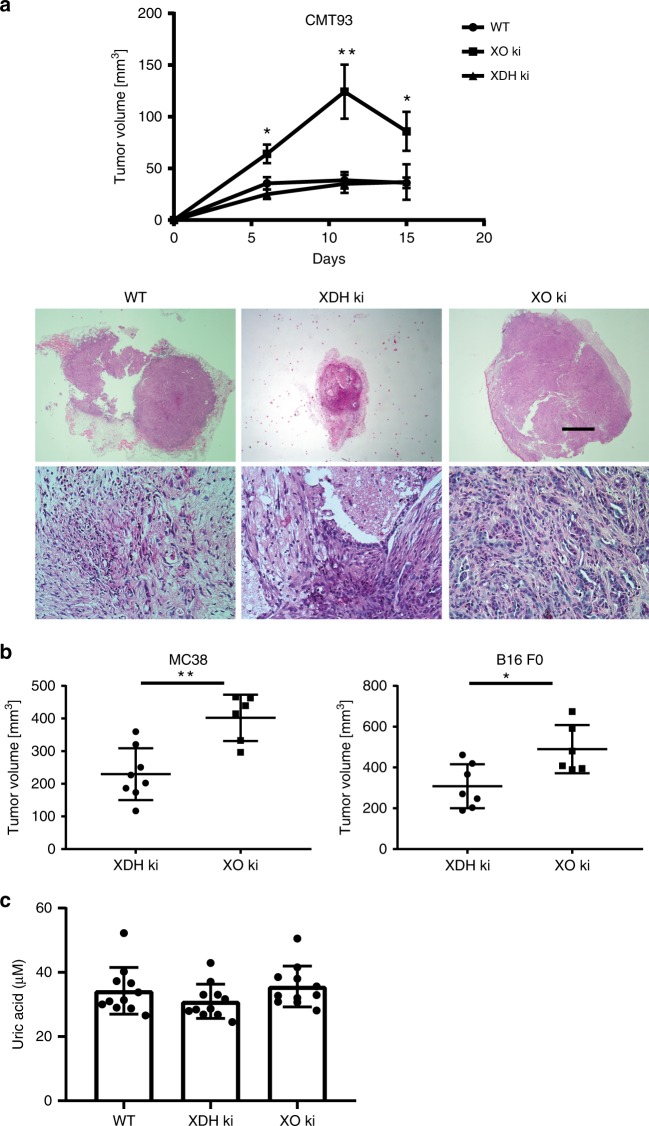
XO ki mice exhibited abnormal tumor growth. **a** CMT93 tumor cells were transferred subcutaneously in the flank of WT, XO ki, and XDH ki mice. Results are expressed as the mean ± SEM of tumor volume from 12 mice. Tumors were resected 15 days after inoculation and subjected to hematoxylin and eosin staining. Representative examples of H&E sections of tumors from WT, XDH ki, and XO ki mice. Images are taken at X40 (top panel, scale bar represents 1 mm) or X400 (bottom panel) magnification. **b** MC38 and B16 F0 melanoma tumor volume in XDH ki and XO ki mice. Data shows the mean ± SEM of tumor volumes from 6 to 8 mice per group. **c** Seric uric acid levels of CMT93 tumor-bearing WT, XDH ki, and XO ki mice at day 15 after tumor transfer. Data show representative mean ± SEM of 11 mice. *P* values were determined by unpaired Student’s *t* test. Difference were considered statistically significant at *p < 0.05, **p < 0.01. Source data are provided in Source Data excel file

**Fig. 4:**
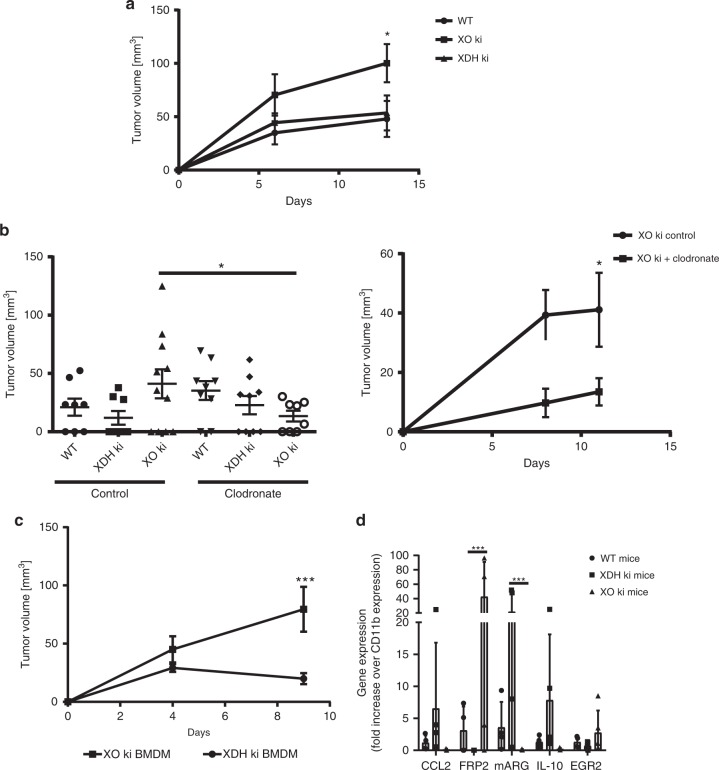
XO ki macrophages are involved in tumor progression. **a** Tumor growth of subcutaneous CMT93 tumors in WT → WT, XDH ki → WT, and XO ki → WT bone marrow chimeric mice (*n* = 10 mice per group). Tumor growth was increased in XO ki → WT mice compared with WT → WT and XDH ki → WT mice. **b** WT, XDH ki, and XO ki mice were injected intraperitoneally with clodronate-loaded liposome suspension prior to tumor injection. CMT93 tumor cells were injected into the flank of mice. Tumor growth was measured using a caliper. Results are expressed as the mean ± SEM of tumor volumes (*n* = 8–11 mice per group). **c** CMT93 tumor growth in WT mice adoptively transferred with XO ki or XDH ki BMDMs. Data shows the mean ± SEM of tumor volumes (4–5 mice per group). **d** Expression levels of M1 and M2 markers in tumors were measured by qRT-PCR and expressed as fold increase over CD11b expression. Results are expressed as the mean ± SEM of RNA extracted from at least 4 mice per group. Difference were considered statistically significant at *p < 0.05, **p < 0.01, *** *p* < 0.001. Source data are provided in Source Data excel file

**Fig. 5:**
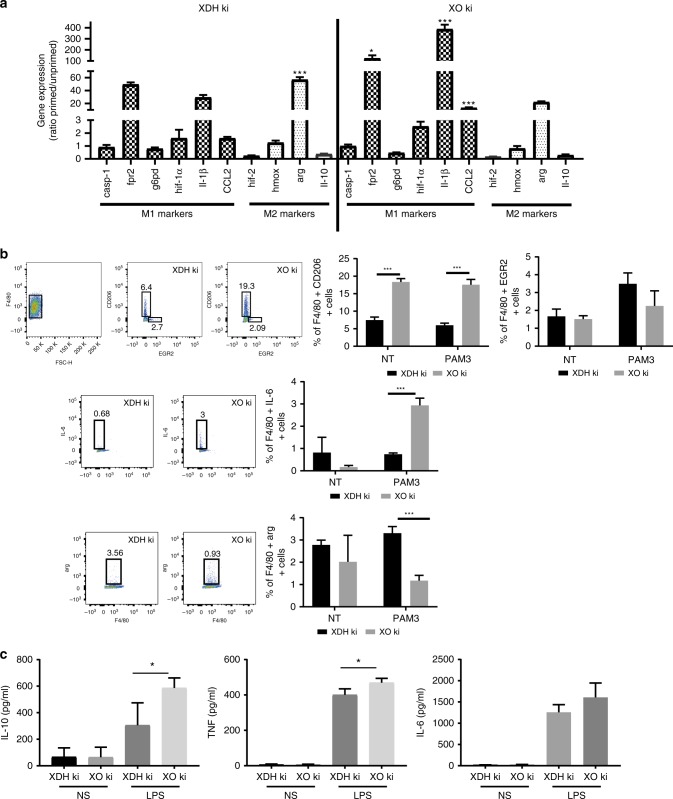
Characterization of XO ki and XDH ki BMDM. **a** XDH ki and XO ki BMDM were primed overnight with or without 100 ng/mL of Pam3CSK4. RT-qPCR analysis of M1/M2 markers expressed as ratio of primed over unprimed cells. Significant increase of gene expression between XO ki and XDH ki macrophages is indicated. **b** Expression of arg, CD206, EGR2, and IL-6 on Pam3CSK4 primed BMDM was analyzed by FACS. **c** XDH ki and XO ki BMDM were primed overnight with or without LPS (100 ng/ml). The supernatants were analyzed for IL-10, TNF, and IL-6 by ELISA. **a**–**c** Results are expressed as the mean ± SEM from differentiated BMDM from at least 3 mice per group. *P* values were determined by *t*-tests. *p < 0.05, **p < 0.01, ***p < 0.001. Source data are provided in Source Data excel file

Two types of XOR mutant knock-in (ki) mice, XDH ki and XO ki, were generated by replacing wild type XOR in C57BL/6 mice (Fig. 1a). W338A/F339L mutations were introduced into exon 11 of the *Xdh* gene in the case of XO ki (Fig. 1b). In the case of the XDH ki, C995R mutation was introduced into exon 27 of the *Xdh* gene (Fig. 1c). The WT *Xdh* locus, construct of targeting vector, and the targeted allele after homologous recombination are depicted in Supplementary Fig. 1A (for XO ki) and S1B (for XDH ki) and further detailed characterization of these knock-in mice is shown in Supplementary data and Figs. 2–5. Homozygous XOR mutant mice were viable, present at the expected Mendelian ratios and did not exhibit overt abnormalities.

**Fig. 6:**
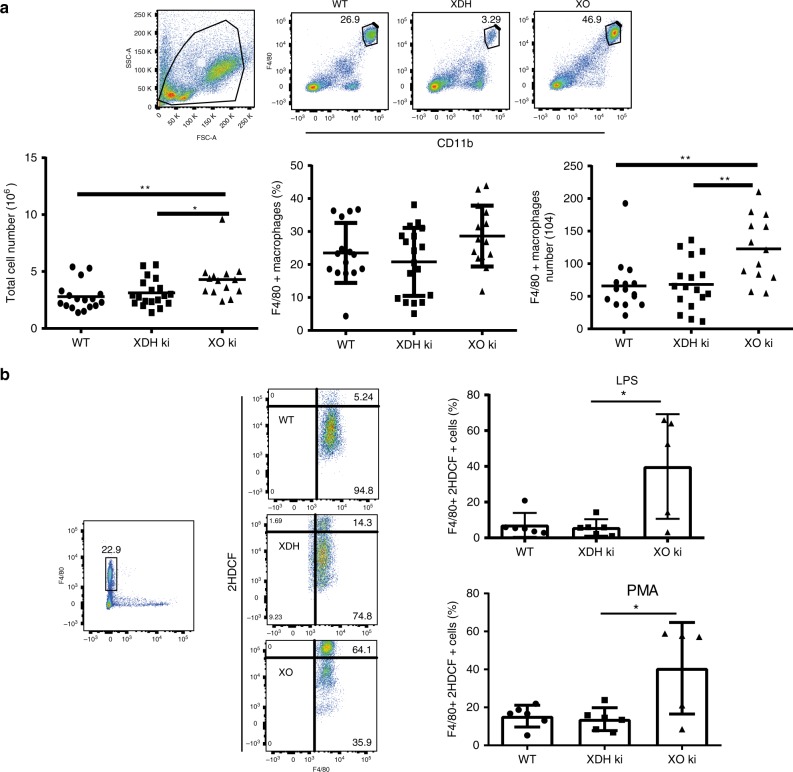
Increased ROS production by peritoneal XO ki macrophages. Peritoneal cells were harvested from WT, XDH ki, and XO ki mice, immunostained and flow cytometry performed to identify F4/80+ macrophages. **a** Total number of PEC from WT and XDH ki and XO ki mice, and F4/80+ macrophages. Results are expressed as the mean ± SEM of percentage of total cells or absolute numbers (*n* = 13–20 mice per group). **b** PEC cells were stimulated ex vivo with LPS or PMA for 3 h. ROS production in F4/80+ macrophages was measured by flow cytometry using CM-DCFH dye. Results are expressed as the mean ± SEM (*n* = 5-6 mice per group). Difference were considered statistically significant at *p < 0.05, **p < 0.01. Source data are provided in Source Data excel file

**Fig. 7:**
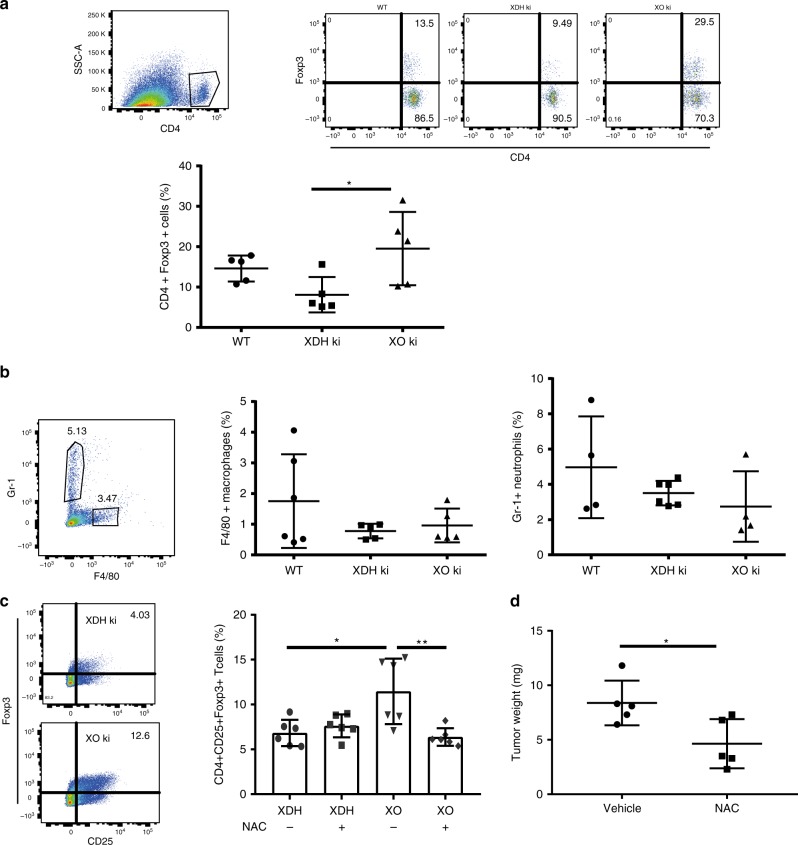
Increased Tregs in XO ki tumors. Tumors were resected 12 days after inoculation and enzymatically digested. **a**, **b** Graphs and FACS profiles indicating the proportions of CD4+Foxp3+ cells, Gr-1+ neutrophils and F4/80+ macrophages in tumors from WT, XDH ki, and XO ki mice. Results are expressed as mean ± SEM from 5 mice. **c** Representative FACS profiles and quantification of CD4+CD25+Foxp3+ T cells in vitro. XDH ki and XO ki BMDMs were cultured with CD4+CD25- T cells and stimulated with OT-II peptide for 5 days in the presence or absence of 1 mM NAC. Results are expressed as mean ± SEM. **d** Tumor weights 9 days after implantation of CMT93 cells in XO ki mice treated with vehicle or NAC at 20 mM in drinking water. Data show the mean ± SEM of tumor weight from 5 mice per group. *P* values were determined by *t*-test. Difference were considered statistically significant at *p < 0.05, **p < 0.01. Source data are provided in Source Data excel file

**Fig. 8:**
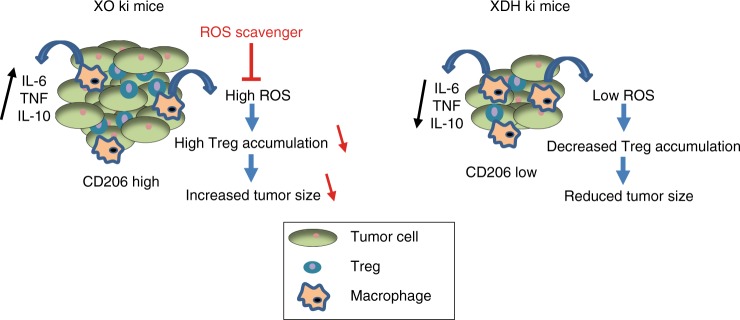
Proposed mechanisms of preferential tumor growth in XO ki mice. In vitro, XO ki macrophages generated more ROS and induce Treg differentiation in a ROS dependent manner. In addition, they produce more inflammatory (IL-1, IL-6, and TNF) and anti-inflammatory (IL-10) cytokines. In vivo, XO ki macrophages establish a tolerogenic microenvironment, increasing Treg accumulation in subcutaneously transferred tumors and thereby favor their growth. A ROS scavenger (NAC) counteracts the pro-tumorigenic phenotype in XO ki mice, reestablishing tumor immunosurveillance (effects of NAC indicated in red)

### Growth and survival of XOR mutant mice

Growth of XO ki and XDH ki was evaluated by body weight gain for up to 19 weeks (Supplementary Fig. 6). There was no difference between the growth of XOR mutant mice and that of WT mice. The estimation of survival distribution for XO ki and XDH ki mice was compared with WT mice by the Kaplan-Meier method (Supplementary Fig. 7). There was no noticeable difference in survival between XOR mutant mice and WT mice.

### Expression of modified XOR protein and activity in ki mice

Expression of mutated XOR in XO ki and XDH ki mice was examined by Western blot analysis of tissue lysate proteins from lung and liver, using anti-rat XOR antibody, which cross-reacts with purified mutated XOR proteins. An intense 150 kD band could be detected in XO ki and XDH ki lung and liver homogenates similar in size and intensity to that in WT counterparts (Supplementary Fig. 8A, B). To assess if targeted XOR mutations could affect XOR activity, we next quantified total XOR activity and XO activity in liver homogenates by assessing xanthine conversion to urate in presence or absence of NAD respectively. In WT liver tissues and XDH stable (C995R) mutant mice, the vast majority of XOR activity was accounted for by XDH activity as XO activity represented <20% of the total XOR activity (Table 1). By contrast in the XO-locked (W338A/F339L) liver homogenates, XO activity was dominantly expressed and no XDH activity could be detected (Table 1). The liver XO activity was not due to proteolytic cleavage since as mentioned above a single 150 kD band was detected in XO ki liver homogenates.

**Table 1:** Comparision of liver lysate XOR activity

	XO ki vs. wild-type		XDH ki vs. wild-type	
	Activity (nmol/min/mg protein)		Activity (nmol/min/mg protein)	
	NAD (−)	NAD (+)	NAD (−)	NAD (+)
Wild-type litter mate	0.64 ± 0.05	3.97 ± 0.11	0.53 ± 0.06	4.10 ± 0.31
Mutant	1.22 ± 0.14	1.19 ± 0.15	0.66 ± 0.14	4.11 ± 0.41

NAD (−), rate of urate formation for xanthine-O_2_ turnover

NAD (+), rate of urate formation for xanthine-NAD^+^ and xanthine-O_2_

Sample number of XO ki mice, wild type litter mate of XO ki, XDH ki mice, and wild type litter mate of XDH ki were 7, 5, 6, and 6, respectively. Activities indicated by mean ± SEM values

### Purification and characterization of XOR from ki mice

XOR of XO ki and XDH ki was purified from the corresponding mouse liver lysates. A single band on SDS-PAGE could be obtained in XO ki (Fig. 2a) and XDH ki (Fig. 2b). We further assessed that the purified XOR (C995R) from XDH ki was resistant to the conversion into the XO form since it lacks the Cys995 involved in the disulfide bridge formation. Indeed, XOR from XDH ki was unable to be converted into XO by chemical modification of the intra-molecular sulfhydryl group with 4, 4′-dithiodipyridine (Fig. 2c). In the same conditions, XDH from bovine milk was converted to XO (Fig. 2d).

Next, we characterized the XO-locked (W338A/F339L) mutant enzyme from XO ki. The purified mutated enzyme generated higher O_2_^–^ levels compared to WT enzyme (Fig. 2e). Altogether, our results indicated that XDH ki expressed an XDH-stable (C995R) mutant protein unable to be converted into XO and that XO ki expressed a XO-locked mutant protein overproducing superoxide.

### XO ki mice exhibit increased tumor growth

XOR has been shown to play a role in tumor growh^20,22,23,30–32^. As the respective roles of XDH and XO and the cells expressing them are not clear in susceptibility to tumor growth, we tested our mice expressing the XO and XDH forms in an in vivo model of subcutaneously transferred CMT93 colon carcinoma cells (expressing WT XOR). In this experimental tumor model, tumors are initially controlled by the adaptive immune response. As shown in Fig. 3a, CMT93 tumor volume reached rapidly a larger size in XO ki mice compared to XDH ki or control WT mice. These clinical observations were confirmed by histological analysis of the dissected tumors, where tumors were consistently bigger in XO ki mice compared to XDH ki and WT mice (Fig. 3a). However, tumor infiltrating T cells and macrophages (shown by anti-CD3 and anti-MAC-2 immunohistology, respectively) and tumor neovascularization (shown by anti-CD31 immunohistology) were not significantly different between mouse genotypes (Supplementary Fig. 9). In addition, we explored the pro-tumor potential effect of the XO form using two other adoptively transferred tumor models, the MC38 colon carcinoma cells and the B16 F0 melanoma cells. The results in Fig. 3b showed that also MC38 tumor grew more aggressively in XO ki mice than in XDH ki mice: 402.1 +/− 29.05 in XO ki vs 229.6 +/− 28.09 in XDH ki mice. Moreover, the average volume of B16 melanoma recovered from the XO ki mice was 60% larger than that from XDH mice: 489.9 +/− 48.37 in XO ki vs 308.2 +/− 40.7 XDH ki mice.

Uric acid may contribute to cancer pathogenisis^33^. To test the role of uric acid in tumor progression, we measured serum uric acid levels in mice after the CMT93 tumor transfer. As shown in Fig. 3c, we found no significant difference between serum uric acid levels in XO ki, XDH ki, and WT mice, thus suggesting that uric acid does not account for increased tumor growth in XO ki mice.

### XO ki macrophages are responsible for faster tumor growth

To investigate if bone-marrow-derived cells or radio-resistant non-hematoipoietic cells are responsible for the increased tumor growth in XO ki mice, we generated chimeric mice by transplantation of bone marrow (BM) from WT, XO ki and XDH ki mice into lethally irradiated WT mice (950 Rad). Chimeric mice were then challenged with tumor cells 8 weeks after the bone marrow transfer (when the adaptive immune system was reestablished). As shown in Fig. 4a, only transfer of XO ki BM to WT mice led to increased tumor growth in these mice thus demonstrating that bone-marrow-derived cells are responsible for the tumor-promoting effect. Amongst hematopoietic cells, we specifically addressed the role of macrophages in increased tumor growth in XO ki, investigating the effects of macrophage depletion on tumor progression. Macrophage depletion was achieved by treating mice with liposomal clodronate, which induced >80% depletion of macrophages by FACS analysis of peritoneal and spleen cells (data not shown). As shown in Fig. 4b, liposomal clodronate treatment inhibited tumor growth in the XO ki mouse group. Next, we sought to examine the pro-tumoral potential of XO ki macrophages. Prior studies have shown that adoptively transferred immune suppressive macrophages stimulate tumor growth^34^. Accordingly, in vitro differentiated XO ki and XDH ki bone-marrow-derived macrophages (BMDM) were injected i.v. into WT mice that had been given CMT93 tumor cells 48 h previously. Remarkably, XO ki BMDM potently stimulated tumor growth compared with XDH ki macrophages in WT mice (Fig. 4c). Taken together; these results indicate the potent ability of XO macrophages in controlling increased tumor growth.

### Characteristics of tumor-associated macrophages in ki mice

As XO ki macrophages were able to favor tumor growth, we hypothesized that they might be phenotypically different compared to XDH ki macrophages. Indeed, tumor-associated macrophages (TAM) can have pro-tumor or anti-tumor activities. Often M1-like (anti-tumor) and M_2_-like (pro-tumor) differentiation were used to classify them, TAM in malignant tumors resemble M2-type macrophages. However, this classification is oversimplified since in certain tumors infiltrating macrophages have a mixed phenotype on a scale in which M1 and M2 represent the extremes^35–37^.

We first proceeded to quantify by qPCR M1 and M2 markers in tumors of XO or XDH ki mice after calibration to CD11b or F4/80 expression. We observed strikingly increased expression of Fpr2 in XO mice, a marker for M1 macrophages and/or inflammation, whereas XDH ki mice expressed higher levels of arginase (arg), a marker for M2 macrophages (Fig. 4d) within the tumor. However, expression of other M1/M2 markers (such as CCL2, IL-10, and EGR2) was similar between XO ki and XDH ki tumor infiltrating macrophages (Fig. 4d).

### Analysis of gene expression in XO ki and XDH ki BMDM

As tumor infiltrating macrophages are heterogenous, we compared the results with in vitro differentiated and activated macrophages. We therefore analyzed M1 and M2 marker expression by RT-qPCR, FACS, and ELISA in activated BMDM from XO and XDH ki mice. We found striking differences between XO ki and XDH ki activated macrophages. First, as found by qPCR, Fpr2 was up-modulated in XO ki activated macrophages whereas arg was down-modulated (Fig. 5a), recapitulating the regulation previously found in tumor infiltrating macrophages (Fig. 4d). In addition, XO ki BMDM expressed more IL-1β and CCL2. By FACS analysis, we found decreased expression of the M2 marker arg in XO ki cells, corroborating the results obtained at the RNA levels, whereas another M2 marker, EGR2 was similarly expressed in both XO ki and XDH ki macrophages. CD206, a mannose receptor and C-type lectin, primary expressed on the surface of M2 macrophages was more abundantly expressed on XO ki BMDM. Finally, XO ki macrophages expressed more IL-6, as demonstrated by FACS (Fig. 5b).

Cytokine levels were also measured by ELISA in supernatants of LPS-stimulated BMDM. We found that XO ki macrophages secreted higher levels of IL-10 and TNF-a than XDH ki macrophages (Fig. 5c).

### Increased ROS production upon XO ki macrophage activation

We assessed by flow cytometry whether the mutant mice had different resident peritoneal cell populations. We found a significant increase in the total peritoneal cell numbers in XO ki mice compared to XDH ki and WT mice (Fig. 6a). To identify specific cell populations in the peritoneal cavity of mice, cells were stained for the macrophage marker F4/80 and neutrophil marker Gr-1. Flow cytometry analysis identified a statistically significant increase in F4/80^+^ macrophage number when comparing the XO ki mice to XDH ki or WT mice (Fig. 6a), whereas the numbers of Gr-1 expressing neutrophils were similar (results not shown). No significant difference appeared between the three groups of mice in the number of macrophage in the spleen and bone marrow (results not shown).

To investigate functional differences in peritoneal macrophages from the different XOR mutant mice, we examined ROS generation upon stimulation of peritoneal macrophage cells. Detection of DCF dye by flow cytometry was used as an indicator of ROS production. As shown in Fig. 5b, LPS or PMA treatment for 3 h led to a significant increase of ROS production in XO ki macrophages when compared with that of LPS or PMA-treated XDH ki or WT macrophages confirming the role of XO as a major source of ROS in these cells.

### Increased Tregs in XO ki tumors

FoxP3^+^ regulatory T cells (Tregs) have a prognostic relevance in cancer, high Treg/CD4 T cell ratios correlate with more aggressive cancers^38^. Interestingly Treg differentiation has been shown to be modulated by ROS^39^. Taking into account the different ROS levels measured in XO ki and XDH ki macrophages, we hypothesized that Treg ratios in intratumoral T cells could vary between XO ki and XDH ki mice. Indeed, flow cytometric analysis of tumor-infiltrating lymphocytes from XO ki mice revealed higher frequencies of Foxp3 regulatory T cell (Fig. 7a). By contrast, no differences could be observed with CD11b^+^ myeloid cells or F4/80^+^ cells (Fig. 7b).

Next, we examined the direct effect of macrophages on Foxp3 expression by CD4^+^ T cells. Differentiated XO ki and XDH ki BMDM were incubated with OTII CD4^+^CD25^−^ T cells at a 1-to-5 ratio in the presence of OTII peptide and TGFbeta and the frequency of Foxp3^+^ Treg cells was examined at day 5 (Fig. 7c). The percentage of FoxP3^+^ cells amongst the CD4^+^CD25^+^ cells was significantly increased upon coculture of T cells with XO ki BMDM, confirming their Treg-inducing capacity. To study if ROS coud be involved in the induction of Treg by XO ki macrophages, we tested the effect of N-Acetyl Cysteine (NAC), a ROS scavenger. XO ki BMDM cultured in the presence of NAC significantly reduced the percentage of Tregs (Fig. 7c), demonstrating the ROS dependency of Treg induction. Based on this observation, we tested the anti-tumoral activity of NAC in vivo. NAC was given into drinking water 24 h after injection of CMT93 tumor cells in XO ki mice. We found that tumor growth was significantly inhibited in the NAC treatment group compared to control mice without NAC treatment (Fig. 7d).

## Discussion

XDH is ubiquitously present in most organisms, from bacteria to human. However, only the mammalian XDH can be converted into the XO conformation, but the pathophysiological reasons underlining the conversion into the XO form remains elusive. The generation and analysis of the *Xdh* gene-modified mice, expressing knocked-in XDH stable (C995R) mutant protein, is an effective method to elucidate the physiological function of dehydrogenase/oxidase conversion. On the other hand, XO is an endogenous source of reactive oxygen species such as hydrogen peroxide and super oxide in vivo. The other *Xdh* gene-modified mice, express knocked-in XO-locked (W338A/F339L) mutant protein. In addition to the physiological role for the purine metabolism, XO-locked mutants can constantly overproduce superoxide in XO ki mouse cells.

These latter mice constitute an unvaluable tool to evaluate the effect of superoxide in vivo.

It is well known that insufficient XOR activity causes serious consequences due to accumulation of high concentration of hypoxanthine and xanthine in blood of most of mammalian animals except primates^8,40^. However, both XDH ki and XO ki mice have a normal phenotype and growth, and in particular no sign of renal failure and premature death, which suggests that XOR mutant enzymes expressed normally and sufficiently with normal physiological function in the XOR gene-modified mice. Indeed, we found that the mutant XOR enzymes were similarly expressed in the lungs and liver of XDH ki, XO ki, and wild-type mice.

Our results questioned a previous publication in which the death of XOR knock-out mice pups was attributed to insufficient milk secretion^8^. It was suggested that the structural changes into the XO form affected lipid secretion in milk. Therefore, deficient breast-feeding ability in XDH ki mice was expected. However, since the growth of XDH ki mice was similar to that of XO ki and WT mice, it seems that even with reduced amounts of XO in milk (such as the situation in lactating XDH ki mice), breast-feeding is not impaired. Phenotypic changes such as early aging and tumorigenesis were expected in XO ki mice, since XO-locked (W338A/F339L) mutant constantly produces superoxide in their cells. Contrary to expectation, these mice did not have any spontaneous phenotype. Growth and life span of XO ki mice were also normal.

Purified XOR enzymes from the XOR gene-modified mice were characterized to confirm that the mutant XOR enzymes have been correctly expressed in vivo. Rat C995R mutant resisted to complete conversion to XO when the enzyme was treated with sulfhydryl-modifying reagent, 4, 4′-dithiodipyridine^41^. Purified XOR from the XDH ki mice resisted to conversion to XO form similar to the property of rat C995R mutant enzyme (Fig. 2). On the other hand, rat W335A/F336L mutant has the XO-locked and superoxide hyper-generation properties^42^. Purified XOR from the XO ki mice constantly exhibited oxidase activity and predominantly generated superoxide similar to the property of rat W335A/F336L mutant enzyme. Altogether, these results confirmed that XDH-stable or XO-locked mutants were successfully introduced into XDH ki or XO ki, respectively.

Several studies have reported a role of the XOR enzyme in cancer. However, surprisingly little is known about the molecular mechanisms underlying the involvement of the two forms of this enzyme in immune pathways and tumorigenesis. Moreover, how and in which cell types an imbalance in oxidase and deydrogenase forms could influence tumor growth is not known. The previous studies concerned XOR expression mostly in cancer cells and both, positive or negative influences of XOR on tumor growth, have been reported^30,43,44^. We therefore here examined the function of our XO and XDH knock-in mice in the syngenic subcutaneously transplanted colorectal cancer model CMT93. In our model, the transplanted tumor cells expressed wild-type XOR activity. The most relevant finding of this study was that mice expressing the XO-fixed form showed strongly enhanced tumor growth when compard to XDH-locked or WT mice.

Cancer infiltrating and systemic myeloid-derived suppressor cells have been associated with enhanced tumor growth. These tumor-derived suppressor cells are a heterogenous mixture of neutrophils, macrophage/monocyte and dendritic cells with varying phenotypes^45^. As in our mice neutrophil marker expressing cells were similar in XO and XDH ki mice, we concentrated on tumor-associated macrophages (TAMs). TAMs have a key role in cancer-related inflammation and immune response/immune escape. TAMs are a heterogenous cell population that can be conventionnally divided into two major subgroups (M1 and M2). While M_2_^−^type macrophages are thought to promote tumor progression, M1-type macrophages are well-adapted to promote a strong immune response and eliminate tumor cells^46^. However, it is important to mention that TAMs can adopt multiple intermediate phenotypes with different features^47^ and that tumors in vivo can express both M1 and M2 markers^48,49^. We established that XO ki macrophage favored tumor progression, based on XO ki bone-marrow transfer experiments or XO ki BMDM injection into wild-type mice, which both promoted tumor growth. Further demonstration was provided by liposomal clodronate treatment which, by depleting macrophages, inhibited tumor growth in the XO ki mouse. We hypothesized that macrophages from XO ki mice were phenotypically different and prone to M2 differentation and pro-tumor activity. We found increased CD206 expression, increased proinflammatory cytokine secretion of IL1β, IL-6, and TNFα, as a consequence of inflammation, increased Fpr2 expression, but also increased secretion of the anti-inflammatory cytokine IL-10 in XO ki macrophages upon activation. There are three possible interpretations for this. First, the regulation of the measured markers and cytokines in XO ki macrophages could be the reflection of macrophage differentiation into mixed M1 and M2 populations. Indeed M1 and M2 macrophages were reported to co-exist inside tumors^48,49^. Secondly, the phenotype of activated XO ki macrophages could reflect XO ki macrophage differentiation towards M2b, a subset of M2 macrophages, which are pro-tumorigenic and express concomitantly CD206, proinflammatory IL1β, IL-6, TNFα, and anti-inflammatory IL-10 cytokines^50^. Finally, the phenotype coud reflect the specific macrophage environment which may alter the gene expression of the different subtypes. It is difficult to discriminate between these possibilities, due to multiple intermediate phenotypes and plasticity observed between M1 and M2 marker expressions^47^.

We speculated that higher ROS production in XO ki macrophages could lead to increased Treg differentiation. Tumors with high Treg/T cell ratios were favorable for tumor growth and predicted poor cancer patient survival^51–53^. Strikingly, ROS have dose-dependent effects on T cells. At low concentrations, ROS are required for effector T cell differentiation, at higher levels, they down-regulate T cell activity and switch the immune response towards Treg phenotype and, at very high concentrations, they become cytotoxic^54,55^. Indeed, we found an increase in Tregs in the fastly growing tumors in XO ki mice compared to XDH ki or WT mice. Also in vitro, XO ki macrophages induced much higher Treg differentiation from naïve T cells. Blocking ROS in vitro and in vivo resulted in reduced Treg formation and reduced cancer growth, respectively. Taken together, these findings provide the link between increased macrophage ROS production, increased Tregs in tumors and increased cancer growth in XO ki mice and were summarized in Fig. 8.

We also investigated whether uric acid, the product of XOR activity, could mediate tumor growth in XO ki mice. It has been reported that UA levels are higher in cancer patients^56^. Interestingly, higher levels of uric acid in tumor-bearing rats were associated with an increase in XO activity^57^. However, in our study, we did not observe any significant differences in uric acid levels between XDH ki, XO ki and WT mice, thus ruling out that this metabolite is responsible for the tumor burden seen in XO ki mice.

Our study also analyzed the Th1 and Th2 cytokine levels in the supernatant of isolated macrophages after in vitro stimulation. Pro-inflammatory Th1 cytokines (IL-12, IFN-γ) have been found to play a major role in inhibiting and killing tumor cells and impeding tumor growth. In contrast, Th2 cytokines have been proposed to facilitate tumor growth and produce anti-inflammatory cytokines such as IL-10^58–60^. Finally, different studies have demonstrated the ability of TNFα to favor tumor escape from immune surveillance^60,61^. In our study, IL-10 and TNFα cytokine levels were found to be up-regulated in the supernatant of XO ki macrophages compared to XDH ki cells. Increase of these cytokines in the tumor microenvironment in XO ki mice may contribute to tumor progression.

Here we show that XO ki and XDH ki macrophages differentially influence the tumor microenvironment, thereby providing a new mechanism by which an imbalance within the enzymatic XO/XDH ratio correlates with an increase of tumor burden. Altogether our results suggest that a macrophage-specific XO inhibitor that would reduce ROS levels but leave XDH activity untouched may prove useful as a cancer therapeutic tool.

## Methods

### Animals

X*dh* gene-modified mice were produced at Nippon Medical School. The mouse knocked-in the XO form (W338A/F339L mutant), was named C57BL/6-*Xdh*^tm1^/Nms. The mouse knocked-in the XDH-stable (C995R mutant) was named C57BL/6-*Xdh*^tm2^/Nms. C57BL/6-*Xdh*^tm1^/Nms and C57BL/6-*Xdh*^tm2^/Nms were abbreviated as XO ki and XDH ki, respectively. The details of the *Xdh* gene-modified mice were described in the supplementary results. WT, XDH ki and XO ki mice, all in the C57BL/6J background, were age and sex-matched in the different experimental settings. WT mice were purchased from Harlan. Unless otherwise stated, mice were used between 8–12 weeks old. All in vivo experimental protocols were approved by the institutional animal care committees of the Nippon Medical School and by the Swiss Federal and Cantonal Veterinary authorities, and throughout the study, extreme care was taken to minimize animal pain and discomfort.

### Materials

*ß*-NAD^+^ (grade V-C), 4,4′-dithiodipyridine (4-DPS) and xanthine were obtained from Sigma-Aldrich (St. Louis, MO). Dithiothreitol (DTT) was purchased from Wako Pure Chemical Industries, Ltd. (Osaka, Japan). All other chemicals and reagents were of reagent grade, or equivalent, and were purchased from Wako Pure Chemical Industries, Ltd. (Osaka, Japan) or Sigma-Aldrich Co. (St. Louis, MO).

### Protein determination

Protein concentration was determined by the dye-binding method of Bradford^62^ with bovine serum albumine as the standard. The concentration of bovine milk XOR and murine liver XOR was determined spectrophotometrically using an extinction coefficient of 37.8 and 35.8 mM^−1^ cm^−1^ at 450 nm, respectively^63,64^.

### XOR activity assay

Enzyme assays were carried out at 25 °C in 50 mM potassium phosphate buffer (pH 7.8) containing 0.4 mM EDTA and 0.15 mM xanthine for XO activity. In the case of total activity (XO and XDH), 500 µM NAD^+^ was added to the reaction. XOR activities (XO activity and total activity) were determined as urate formation rate by monitoring the absorbance change at 295 nm. The extinction coefficient of 9.6 mM^−1^ cm^−1^ for urate was used. Uric acid and O_2_^−^ quantification during xanthine-O_2_ turnover was described previously^29^. Photometric experiments were performed with a UV-2550 spectrophotometer (Shimadzu, Kyoto, Japan). The cell holder was water-jacketed and maintained at a constant temperature of 25.0 °C.

### Electrophoresis

Sodium dodecyl sulfate-polyacrylamide gel electrophoresis (SDS-PAGE) on slab gels was performed by using a polyacrylamide gradient gel (Mini-PROTEAN TGX 5–20%, Bio-Rad Laboratories Inc., Hercules, CA), according to the method of Laemmli^65^. Mixture of recombinant proteins (Precision Plus Protein Standards, Bio-Rad) was used as a molecular weight standard marker. Separated proteins by SDS-PAGE were stained with fluorescent dye (Oriole fluorescent gel stain, Bio-Rad) and were visualized by a gel imaging system (ChemiDoc XRS+, Bio-Rad).

### Western blotting analysis

Proteins resolved on a SDS-PAGE gel were blotted by electrophoretic transfer into a PVDF membrane using the Trans-Blot Turbo Blotting System (Bio-Rad). Western blots were washed with 0.05 M Tris-HCl (pH 7.6), 0.15 M NaCl, 0.5% Tween 20 (TBS-T) for 5 min, and blocked for 1 h in 5% (w/v) skimmed milk in TBS-T at ambient temperature. A rabbit polyclonal antibody against rat XOR (laboratory-prepared antibody) and HRP conjugated goat polyclonal anti-rabbit antibody (1:2000, DAKO Cytomation, Glostrup, Denmark) was used as a primary antibody and secondary antibody, respectively. The cross-react signals were developed using the ECL Prime reagent (GE Healthcare UK Ltd.) and were visualized by a gel imaging system (ChemiDoc XRS+, Bio-Rad).

### Purification of XOR

Quality of the purified XOR enzymes were evaluated by using activity flavin ratio (AFR)^66^. AFR was obtained by dividing the change in absorbance per min at 295 nm by the absorbance at 450 nm of the XOR enzyme used in the assay. In the case of fully active XOR, AFR is around 200. The two mutants XOR, XO ki (W338A/F339L), and XHD ki (C995R) were purified from mice liver as described previously^67^; but using only the first affinity chromatography due to limited amount of enzyme availability. Briefly, ammonium sulfate fraction from liver soluble fraction was applied to an anion exchange column and a calcium phosphate column. Bovine milk XOR (AFR more than 150) was prepared according to the procedure of Nishino et al.^68^; but using only the first affinity chromatography. The XDH and XO enzyme forms were treated with 5 mM DTT or 0.1 mM 4-DPS for 1 h at 25 °C, respectively, and then passed through a sephadex G-25 column (GE Healthcare Bio-Science Corp. Piscataway, NJ) to remove excess DTT and 4-DPS. The D/O ratio as defined by Waud and Rajagopalan^69^ was determined as the ratio of the absorbance change at 295 nm under aerobic conditions in the presence of NAD to that in the absence of NAD. The bovine milk XDH and the murine liver XDH samples used in this experiment had D/O ratio of more than 7 and 5, respectively.

### Preparation of bone-marrow-derived macrophages (BMDMs)

8–10-week-old mice were euthanized and bone marrow cells were obtained from femurs by flushing the bone marrow cavities with complete media (IMDM, 10% FCS, 0.5% glutamax, 50 U/ml of penicillin, and 50 mg/ml streptomycin, and 20% L929 supernatant (source of M-CSF)). Bone marrow cells were cultured for 3 days in the complete medium in an incubator at 37 °C and 5% CO_2_. On the third and fifth days, the medium was changed. After 7 days, the cells were detached and re-plated at a density of 0.5 × 10^6^ cells per 24-well culture dish. BMDMs cells were treated overnight with 100 ng/ml LPS or with 100 ng/mL of Pam3CSK4 (Invitrogen). Culture supernatants were collected and total RNA or protein lysates were prepared from the cells. All samples were stored at −80 °C until analysis. The purity of the differentiated macrophages were verified by flow cytometry based on their positive staining for F4/80 and CD11b.

### Resident peritoneal exudate cells (PEC)

Euthanized mice were injected i.p. with 3 mL of PBS, the abdomen was gently massaged for 60 s and then cells were harvested, counted, and and resuspended in RPMI 1640 medium, supplemented with 10% FCS.

### Flow cytometric analysis

Cells were resuspended in fluorescence-activated cell sorting (FACS) buffer (5% fetal calf serum plus 5 mM EDTA in PBS) and incubated with conjugated monoclonal antibodies (mAb). The mAb used, phycoerythrin (PE)-conjugated anti–Ly-6G (clone RB6-8C5, 125931, 1/400), fluorescein isothiocyanate (FITC)-conjugated anti-CD11b (clone M1/70, 11011241, and 1/200), and allophycocyanin (APC)-conjugated anti-F4/80 (clone BM8, 17480182, 1/800), eF450-conjugated anti-CD4 (clone RM4-5, 48004282, 1/200), PE-conjugated anti–Foxp3 (clone FJK‐16 s, 12577382, 1/100), FITC-conjugated anti-CD25 (clone PC61.5, 53025382, 1/400), APC-conjugated anti-Arginase-1 (clone A1exF5, 17369782, 1/100), APC-conjugated anti-EGR2 (clone erongr2, 17669182, 1/100), were all from eBioscience. PE-conjugated anti-CD206 (clone CD68C2, 141705, 1/100) and PE-conjugated anti-IL-6 (clone MP5-20F3, 504504, 1/100) were from Biolegend. Cells (1 × 10^6^) were incubated with appropriate conjugated antibodies for 30 min at 4 °C in the dark. Stained cells were subsequently washed twice with FACS buffer and fixed in BD CellFIX solution (BD Biosciences). Flow cytometric analyses were performed on LSRII cytometer (Becton Dickinson) using FACS Diva6 (Becton Dickinson) and FlowJoX (Tree Star) software for data processing.

### ELISA

Supernatant samples were thawed and analyzed for secreted IL-10, IL-6, and TNF-α by ELISA (eBioscience), according to the manufacturer’s protocol. Plates were read at 450 nm in an ELISA reader (SpectraMAX 190 microplate reader).

### ROS measurement

Peritoneal exudate cells were cultured in 24-well plates (0.5 × 10^6^ cells per well) and incubated with LPS (100 ng/ml) or PMA (100 ng/ml). The dye 2′, 7′-dichlorofluorescein diacetate (DCFH-DA, 10 μM) was added and incubated in the dark at 37°C for 3 h. Cells were then washed twice with PBS and the intensity of dichlorofluorescein (DCF) fluorescence in the cell was measured by FACS.

### Tumor experiments

CMT93 (ATCC, CCL-223) and MC38 (Kerafast, ENH204-FP) colon carcinoma tumor cell lines and the B16 F0 melanoma cell line (ATCC, CRL-6322) were cultured in DMEM medium supplemented with 10% FCS, and 50 U/ml of penicillin and 50 mg/ml streptomycin. Cell lines were tested negative for mycoplasma. Tumors were induced by s.c. injection of 2 × 10^6^ CMT93, MC38, or B16 F0 cells into the flank of mice. In one experiment, XO ki or XDH ki BMDMs (2 × 10^6^ cells per mouse) were adoptively transferred by i.v. injection to WT mice 24 h after CMT93 tumor cell implantation. Tumor growth was followed by measuring length and width using a caliper. Tumor volume was calculated using the formula *V* = π 6⋅1.58⋅(length⋅width) as described by Wurzenberger et al.^70^.

### Immunohistological analysis of tumors

At the end of the experiment, tumors were dissected and paraffin embedded. Sections (5 μm) were stained with hematoxylin and eosin, and T cells, macrophage and endothelial cells staining performed by immunohistochemistry using anti-CD3 (Abcam, ab16669, 1/100) anti-Mac-2 (Cedarlane, CL8942AP, 1/20) or anti-CD31 (Abcam, 28364, 1/200) antibodies, respectively. Stainings were graded independently by two observers unaware of mice genotype using a scoring system based on the severity of cell infiltration (for T and macrophage cells) or the number of vessels counted per field (for endothelial cells).

### Bone marrow chimeras

Recipient male mice at 4 weeks of age were lethally irradiated with 950 rads using an X-ray irradiator. Twenty-four hours post irradiation, bone marrow cells were obtained from femurs and tibia of donor mice (WT, XDH ki and XO ki mice), and 10^7^ bone marrow cells were injected i.v. into irradiated recipient mice. Two month later, CMT93 tumor cells were injected s.c. into the flank of mice. Tumor growth was followed by measuring the length and width using a caliper as described above.

### Gene expression analysis by real-time PCR

RNA was extracted from tumors or BMDM. The qRT-PCR was performed using SYBR Green mix on LightCycler480 (384-well plate, 5 μL reaction) from Roche Diagnostics. The following primers were used:

IL-10, F 5′-GCCTTATCGGAAATGATCCA-3′, R 5′-CTCCACTGCCTTGCTC-3′; FPR2, F 5′-CACAGCAACTCTACCATTC-3′, R 5′-CAACCACCTTCCTAGCCA-3′; EGR2, F 5′-ACACTACACCAGCAACTC-3′, R 5′-ACCACAACACATCCACACA-3′; CCL2, F 5′-CATCCACTACCTTTTCCAC-3′, R 5′-GATCCACACCTTGCATTT-3′; Arginase: F 5′-TGAGAGACCACGGGGACCTG-3′, R 5′-GCACCACACTGACTCTTCCATTC-3′; CD11b, F 5′-GAATGCTGCGAAGATCCTAGTTGTC-3′, R 5′-CGGGACTGTGGTTTGTTGAAGG-3′; F4/80, F 5′-GCCTATTATCTATACCCTCCAGCACATC-3′, R 5′-TCCATCTCCCATCCTCCACATCAG- 3; Granzyme B: 5′-AAGAACAGGAGAAGACCCA-3′ and 5′-AACCAGCCACATAGCACA-3′; Hif1α: 5′-ACCTTCATCGGAAACTCCAAAG-3′ and 5′-CTGTTAGGCTGGGAAAAGTTAGG-3′; G6PD: 5′-GGTAAAGGGGTGGTCTGATGG-3′ and 5′-TGCGGGATACAAAGGGATGG-3′; IL-1β: 5′-CAACAAACAAGTGATATTCTCCATG-3′ and 5′-GTGCCGTCTTTCATTACACAG-3′; Casp-1: 5′-CCGTGGAGAGAAACAAGGAG-3′ and 5′-ATGAAAAGTGAGCCCCTGAC-3′; Hmox1 5′-GCCTCCAGAGTTTCCGCATA-3′ and 5′-AGGAAGCCATCACCAGCTTA-3′; Hif2α: 5′-CAGCAAGGAGACGGA-3′ and 5′-GCAATGAACCCTCCAAG-3′. The qRT-PCR was performed with technical triplicates. For the analysis, expression of each gene was normalized to the house-keeping gene β-actin, F 5-GCACAGCTTCTTTGCAGCTCCTTCG-3′, and R 5-TTTGCACATGCCGGAGCCGTTG-3′. Relative change in mRNA expression was calculated using the qBasePlus software (Biogazelle).

### In vivo macrophage depletion

Liposome-encapsulated clodronate (dichloromethylene bisphosphonate) or liposome-PBS were provided by LIPOSOMA, Amsterdam, Netherlands, and were prepared according to the manufacturer’s protocol. Mice were daily injected intraperitoneally with 200 μL of a clodronate-loaded liposome suspension 4 times starting 3 days before tumor injection. Control mice were injected with 200 μL PBS-loaded liposomes using the same schedule.

### Determination of serum uric acid levels

Serum uric acid levels were measured by using a quantitative enzymatic assay (Amplex Red kit, Invitrogen) according to the manufacturer’s protocol. The results were standardized by using a commercial uric acid standard solution.

### In vitro differentiation of regulatory T cells

Total CD4^+^CD25^−^ T cells from spleen of OT-II mice were prepared using regulatory T cell isolation kit (Miltenyi Biotec). Isolated CD4^+^CD25^−^ T cells (10^5^ cells/well) were co-cultured with their corresponding XO ki or XDH ki BMDMs (2.10^4^ cells/well) in 200 μl/well of complete media plus 10 μM of OT-II peptide and 2 ng/ml TGFβ) at 37 °C for 5 days. Differentiation of regulatory T cells was determined by FACS using Foxp-3 staining kit (eBioscience).

### N-Acetyl Cysteine (NAC) treatment

In vitro, NAC (Sigma) was added at 1 mM to cell culture. In vivo, NAC was administered at 20 mM into the drinking water for 24 h after tumor transfer until endpoint.

### Graphical representation and statistical analysis

For in vitro experiments, values represent mean ± SEM of triplicates from one representative experiment of at least two independent experiments. For in vivo experiments, at least 5 mice per group were used. Data were analyzed with GraphPad Prism 8.0 program. Variation between data sets were evaluated using the Student’s *t* test or one-way Anova, where appropriate. Difference were considered statistically significant at *p < 0.05, **p < 0.01, ***p < 0.001.

### Reporting summary

Further information on research design is available in the Nature Research Reporting Summary linked to this article.

## Supplementary information

Supplementary Information

Reporting Summary

Source Data

## Data Availability

All data generated or analyzed during this study are available from the authors. The source data underlying all figures are provided in as a Source Data file (Supplementary Data 1). A reporting summary for this article is available as a Supplementary Information file.

## References

[1] Nishino T (1994). The conversion of xanthine dehydrogenase to xanthine oxidase and the role of the enzyme in reperfusion injury. J. Biochem..

[2] Hille R, Nishino T (1995). Flavoprotein structure and mechanism. 4. Xanthine oxidase and xanthine dehydrogenase. FASEB J..

[3] Nishino T, Okamoto K (2000). The role of the [2Fe-2s] cluster centers in xanthine oxidoreductase. J. Inorg. Biochem..

[4] Hille R (1996). The mononuclear molybdenum enzymes. Chem. Rev..

[5] McCord JM (1985). Oxygen-derived free radicals in postischemic tissue injury. N. Engl. J. Med.

[6] Harrison R (2002). Structure and function of xanthine oxidoreductase: where are we now?. Free Radic. Biol. Med.

[7] Berry CE, Hare JM (2004). Xanthine oxidoreductase and cardiovascular disease: molecular mechanisms and pathophysiological implications. J. Physiol..

[8] Vorbach C, Scriven A, Capecchi MR (2002). The housekeeping gene xanthine oxidoreductase is necessary for milk fat droplet enveloping and secretion: gene sharing in the lactating mammary gland. Genes Dev..

[9] Cantu-Medellin N, Kelley EE (2013). Xanthine oxidoreductase-catalyzed reactive species generation: a process in critical need of reevaluation. Redox Biol..

[10] Waud WR, Rajagopalan KV (1976). The mechanism of conversion of rat liver xanthine dehydrogenase from an NAD^+^-dependent form (type D) to an O_2_^−^dependent form (type O). Arch. Biochem. Biophys..

[11] Nakamura M, Yamazaki I (1982). Preparation of bovine milk xanthine oxidase as a dehydrogenase form. J. Biochem..

[12] Corte ED, Stirpe F (1972). The regulation of rat liver xanthine oxidase. Involvement of thiol groups in the conversion of the enzyme activity from dehydrogenase (type D) into oxidase (type O) and purification of the enzyme. Biochem. J..

[13] Stirpe F, Della Corte E (1969). The regulation of rat liver xanthine oxidase. Conversion in vitro of the enzyme activity from dehydrogenase (type D) to oxidase (type O). J. Biol. Chem..

[14] Nishino T, Nishino T (1997). The conversion from the dehydrogenase type to the oxidase type of rat liver xanthine dehydrogenase by modification of cysteine residues with fluorodinitrobenzene. J. Biol. Chem..

[15] Enroth C (2000). Crystal structures of bovine milk xanthine dehydrogenase and xanthine oxidase: structure-based mechanism of conversion. Proc. Natl Acad. Sci. USA.

[16] Saito T, Nishino T (1989). Differences in redox and kinetic properties between NAD-dependent and O_2_^−^dependent types of rat liver xanthine dehydrogenase. J. Biol. Chem..

[17] Hunt J, Massey V, Dunham WR, Sands RH (1993). Redox potentials of milk xanthine dehydrogenase. Room temperature measurement of the FAD and 2Fe/2S center potentials. J. Biol. Chem..

[18] Chiricolo M, Tazzari PL, Abbondanza A, Dinota A, Battelli MG (1991). Cytotoxicity of, and DNA damage by, active oxygen species produced by xanthine oxidase. FEBS Lett..

[19] Nomura J (2014). Xanthine oxidase inhibition by febuxostat attenuates experimental atherosclerosis in mice. Sci. Rep..

[20] Shmarakov IO, Marchenko MM (2008). [Xanthine oxidase activity in the rat liver tissue in the process of oncogenesis]. Ukr. Biokhim Zh . (1999).

[21] Fini MA (2008). Migratory activity of human breast cancer cells is modulated by differential expression of xanthine oxidoreductase. J. Cell Biochem..

[22] Cantu-Medellin N, Kelley EE (2013). Xanthine oxidoreductase-catalyzed reactive species generation: A process in critical need of reevaluation. Redox Biol..

[23] Prajda N, Morris HP, Weber G (1976). Imbalance of purine metabolism in hepatomas of different growth rates as expressed in behavior of xanthine oxidase (EC 1.2.3.2). Cancer Res..

[24] Linder N (2005). Down-regulated xanthine oxidoreductase is a feature of aggressive breast cancer. Clin. Cancer Res..

[25] Fini MA, Monks J, Farabaugh SM, Wright RM (2011). Contribution of xanthine oxidoreductase to mammary epithelial and breast cancer cell differentiation in part modulates inhibitor of differentiation-1. Mol. Cancer Res..

[26] Huang CC, Chen KL, Cheung CH, Chang JY (2013). Autophagy induced by cathepsin S inhibition induces early ROS production, oxidative DNA damage, and cell death via xanthine oxidase. Free Radic. Biol. Med..

[27] Battelli MG, Polito L, Bortolotti M, Bolognesi A (2016). Xanthine Oxidoreductase-derived reactive species: physiological and pathological effects. Oxid. Med. Cell Longev..

[28] Amaya Y (1990). Proteolytic conversion of xanthine dehydrogenase from the NAD-dependent type to the O2-dependent type. Amino acid sequence of rat liver xanthine dehydrogenase and identification of the cleavage sites of the enzyme protein during irreversible conversion by trypsin. J. Biol. Chem..

[29] Terao M (1992). Molecular cloning of a cDNA coding for mouse liver xanthine dehydrogenase. Regulation of its transcript by interferons in vivo. Biochem. J..

[30] Linder N (2006). Decreased xanthine oxidoreductase is a predictor of poor prognosis in early-stage gastric cancer. J. Clin. Pathol..

[31] Huang CC, Chen KL, Cheung CHA, Chang JY (2013). Autophagy induced by cathepsin S inhibition induces early ROS production, oxidative DNA damage, and cell death via xanthine oxidase. Free Radic. Biol. Med..

[32] Battelli MG, Musiani S, Tazzari PL, Stirpe F (2001). Oxidative stress to human lymphocytes by xanthine oxidoreductase activity. Free Radic. Res..

[33] Fini MA, Elias A, Johnson RJ, Wright RM (2012). Contribution of uric acid to cancer risk, recurrence, and mortality. Clin. Transl. Med..

[34] Kaneda MM (2016). Macrophage PI3Kγ drives pancreatic ductal adenocarcinoma progression. Cancer Discov..

[35] Sica A, Mantovani A (2012). Macrophage plasticity and polarization: in vivo veritas. J. Clin. Invest..

[36] Noy R, Pollard JW (2014). Tumor-associated macrophages: from mechanisms to therapy. Immunity.

[37] Murray PJ (2014). Macrophage activation and polarization: nomenclature and experimental guidelines. Immunity.

[38] Shitara K, Nishikawa H (2018). Regulatory T cells: a potential target in cancer immunotherapy. Ann. NY Acad. Sci..

[39] Kraaij MD (2010). Induction of regulatory T cells by macrophages is dependent on production of reactive oxygen species. Proc. Natl Acad. Sci. USA.

[40] Ohtsubo T (2009). Xanthine oxidoreductase depletion induces renal interstitial fibrosis through aberrant lipid and purine accumulation in renal tubules. Hypertension.

[41] Nishino T (2005). Mechanism of the conversion of xanthine dehydrogenase to xanthine oxidase: identification of the two cysteine disulfide bonds and crystal structure of a non-convertible rat liver xanthine dehydrogenase mutant. J. Biol. Chem..

[42] Asai R (2007). Two mutations convert mammalian xanthine oxidoreductase to highly superoxide-productive xanthine oxidase. J. Biochem..

[43] Battelli MG, Bolognesi A, Polito L (2014). Pathophysiology of circulating xanthine oxidoreductase: New emerging roles for a multi-tasking enzyme. Bba-Mol. Basis Dis..

[44] Casey SC (2015). The effect of environmental chemicals on the tumor microenvironment. Carcinogenesis.

[45] Veglia F, Perego M, Gabrilovich D (2018). Myeloid-derived suppressor cells coming of age. Nat. Immunol..

[46] Mantovani A, Sozzani S, Locati M, Allavena P, Sica A (2002). Macrophage polarization: tumor-associated macrophages as a paradigm for polarized M2 mononuclear phagocytes. Trends Immunol..

[47] Mosser DM, Edwards JP (2008). Exploring the full spectrum of macrophage activation. Nat. Rev. Immunol..

[48] Edin S (2012). The distribution of macrophages with a M1 or M2 phenotype in relation to prognosis and the molecular characteristics of colorectal cancer. PLoS ONE.

[49] Algars A (2012). Type and location of tumor-infiltrating macrophages and lymphatic vessels predict survival of colorectal cancer patients. Int J. Cancer.

[50] Roszer T (2015). Understanding the Mysterious M2 Macrophage through Activation Markers and Effector Mechanisms. Mediators Inflamm..

[51] Chaudhary, B. & Elkord, E. Regulatory T Cells in the tumor microenvironment and cancer progression: role and therapeutic targeting. *Vaccines (Basel)*. **4**, E28 (2016). 10.3390/vaccines4030028 PMC5041022 27509527

[52] Curiel TJ (2004). Specific recruitment of regulatory T cells in ovarian carcinoma fosters immune privilege and predicts reduced survival. Nat. Med..

[53] Nishikawa H, Sakaguchi S (2014). Regulatory T cells in cancer immunotherapy. Curr. Opin. Immunol..

[54] Kienhofer D, Boeltz S, Hoffmann MH (2016). Reactive oxygen homeostasis - the balance for preventing autoimmunity. Lupus.

[55] Roberts CA, Dickinson AK, Taams LS (2015). The interplay between Monocytes/Macrophages and CD4(+) T Cell subsets in rheumatoid arthritis. Front Immunol..

[56] Shin HS (2006). Uric acid as a prognostic factor for survival time: a prospective cohort study of terminally ill cancer patients. J. Pain. Symptom Manag..

[57] Springer J (2012). Inhibition of xanthine oxidase reduces wasting and improves outcome in a rat model of cancer cachexia. Int J. Cancer.

[58] Mocellin S, Marincola F, Rossi CR, Nitti D, Lise M (2004). The multifaceted relationship between IL-10 and adaptive immunity: putting together the pieces of a puzzle. Cytokine Growth Factor Rev..

[59] Grivennikov SI, Greten FR, Karin M (2010). Immunity, inflammation, and cancer. Cell.

[60] Oft M (2014). IL-10: master switch from tumor-promoting inflammation to antitumor immunity. Cancer Immunol. Res..

[61] Suganuma M (1999). Essential role of tumor necrosis factor alpha (TNF-alpha) in tumor promotion as revealed by TNF-alpha-deficient mice. Cancer Res..

[62] Bradford MM (1976). A rapid and sensitive method for the quantitation of microgram quantities of protein utilizing the principle of protein-dye binding. Anal. Biochem..

[63] Johnson JL, Waud WR, Cohen HJ, Rajagopalan KV (1974). Molecular basis of the biological function of molybdenum. Molybdenum-free xanthine oxidase from livers of tungsten-treated rats. J. Biol. Chem..

[64] Massey V, Brumby PE, Komai H (1969). Studies on milk xanthine oxidase. Some spectral and kinetic properties. J. Biol. Chem..

[65] Laemmli UK (1970). Cleavage of structural proteins during the assembly of the head of bacteriophage T4. Nature.

[66] Bray RC, Barber MJ, Dalton H, Lowe DJ, Coughlan MP (1975). Iron-sulphur systems in some isolated multi-component oxidative enzymes. Biochem. Soc. Trans..

[67] Ikegami T, Nishino T (1986). The presence of desulfo xanthine dehydrogenase in purified and crude enzyme preparations from rat liver. Arch. Biochem. Biophys..

[68] Nishino T, Nishino T, Tsushima K (1981). Purification of highly active milk xanthine oxidase by affinity chromatography on Sepharose 4B/folate gel. FEBS Lett..

[69] Waud WR, Rajagopalan KV (1976). Purification and properties of the NAD+-dependent (type D) and O2-dependent (type O) forms of rat liver xanthine dehydrogenase. Arch. Biochem. Biophysics.

[70] Wurzenberger C (2009). Short-term activation induces multifunctional dendritic cells that generate potent antitumor T-cell responses in vivo. Cancer Immunol. Immunother..

