# Supplementary material for: Targeted knock-in mice expressing the oxidase-fixed form of xanthine oxidoreductase favor tumor growth
Source: Nat Commun. 2019 Oct 28;10:4904. doi: 10.1038/s41467-019-12565-z (PMC6817904; doi:10.1038/s41467-019-12565-z)
Supplement: Supplementary file 1 — Supplementary Information [file 41467_2019_12565_MOESM1_ESM.pdf]

# Supplementary Informations

**Teruo Kusano et al.**

**Targeted knock-in mice expressing oxidase fixed form of xanthine oxidoreductase favor tumor growth**

## Supplementary Results

Correct homologous recombination was confirmed by Southern blot analysis. Supplementary Figure 2 and 4 show the results of southern blot identification of W338A/F339L replacement and C995R replacement, respectively. Supplementary Figure 2 shows that the DNA fragments having the 6.8 kbp with Hind III digestion and the 13.3 kbp with EcoR V digestion were detected with 5' and 3' probe, respectively. In Figure S4, the 9.3 kbp fragment with Spe I digestion and 14.3 kbp fragment with Pvu II digestion were detected with 5' and 3' probe, respectively. These results indicated that the targeted allele was transmitted into the germline, and non-specific insertion of targeting vector into mice genome did not occur in both XOR mutant mice. Finally, the neomycin resistance gene was removed through the activity of Cre recombinase acting on the loxP sites after gene targeting (Supplementary Figure 3 and 5).

## Supplementary Methods

C*onstruction of XO ki* – XO ki was produced replacing W338 and F339 in mouse XOR (corresponding to W335 and F336 in rat XOR) with alanine and leucine, respectively (Supplementary Figure 1A). The mutations were introduced into exon 11 of mouse *Xdh* gene (Supplementary Figure 1A). Targeting vector for W338A/F339L mutation (Supplementary Figure 1B) was linearized with Sac II digestion, and then it was introduced into C57BL/6 embryonal stem cells by electroporation (200V, 800μF, Low Ω, CELL-PORATOR, LIFE TECHNOLOGIES). The transformed cells were selected in ES medium containing G418. Clones showed evidence for homologous recombination were injected into Balb/c blastocysts using standard techniques. The obtained chimeric mice that transmitted the mutant allele were mated to C57BL/6 females for germline transmission. Genomic DNA isolated from F1 mouse by tail biopsy was used for genotype screening by Southern blot analysis (Supplementary Figure 2). Heterozygous mice were then interbred to produce homozygous mice. The obtained XO ki-$Neo^r$ mice were crossbred with Cre recombinase expression mice for the purpose of removing a Neomycin resistant gene. Δ*Neo*$^r$ mice resulting from this process were backcrossed with the C57BL/6 wild type mouse to remove *Cre* recombinase gene (Supplementary Figure 3).

C*onstruction of XDH ki* – XDH ki was produced replacing C995 in mouse XOR (corresponding to C992 in rat XOR) with arginine (Supplementary Figure 4A). The mutation was introduced into exon 27 of mouse *Xdh* gene (Supplementary Figure 4A). Targeting vector for C995R mutation (Supplementary Figure 4B) was linearized with Sac II digestion, and then it was introduced into C57BL/6 embryonal stem cells by electroporation (200V, 800μF, Low Ω, CELL-PORATOR, LIFE TECHNOLOGIES). The transformed cells were selected in ES medium containing G418 and gancyclovir. Clones showed evidence for homologous

recombination were injected into Balb/c blastocysts using standard techniques. Healthy injectioned blastocysts were transferred to the pseudopregnant ICR female recipients to obtain chimeric mice. The obtained chimeric males with more than 50% coat color contribution were mated to C57BL/6 females for germline transmission. Genomic DNA isolated by tail biopsy was used for genotype screening by Southern blot analysis (Supplementary Figure 5). Heterozygous mice were then interbred to produce homozygous mice. The obtained XDH ki-Neo$^r$ mice were crossbred with Cre recombinase expression mice for the purpose of removing a Neomycin resistant gene. Δ*Neo*$^r$ mice resulting from this process were backcrossed with the C57BL/6 wild type mouse to remove Cre recombinase gene (Supplementary Figure 6).

*Southern blot analysis for XO ki-Neo$^r$* – Genomic DNA was digested with *Hind* III or *Eco*R V, electrophoresed on an agarose gel, then blotted onto a nylon membrane (GE healthcare) by VacuGene XL (GE healthcare). For 5' probe, 316 bp DNA fragment was amplified with forward primer (5'-GTATTTGGGTACTAAGAGGAACACAAT-3') and reverse primer (5'-AGTGTGTCACAATGCTATAGATGAAGG-3') by PCR. For 3' probe, 294 bp DNA fragment was amplified with forward primer (5'-TACTCTGCTATATTGGCCTAGCACT-3') and reverse primer (5'-ATCCTAGCTCATTCCTATGCTTTTC-3') by PCR. Hybridization and signal detection was performed using an AlkPhos Direct Labeling Reagent (GE healthcar) in accordance with the manufacturer's instructions.

*Southern blot analysis for XDH ki-Neo$^r$* – Genomic DNA was digested with *Pvu* II or *Spe* I, electrophoresed on an agarose gel, then blotted onto a nylon membrane (GE healthcare) by VacuGene XL (GE healthcare). For 5' probe, 297 bp DNA fragment was amplified with forward primer (5'-TTAAAAAGCAGGTTTCCTGTTATGC-3') and reverse primer (5'-TTCTCCCATGCATTGTGTTTATAG-3') by PCR. For 3' probe, 294 bp DNA fragment was

amplified with forward primer (5'-AGTATTCGAGTACCTGGGAATAAGC-3') and reverse primer (5'-CTTCTCCAGCTTGACCTATTCTATG-3') by PCR. Hybridization and signal detection was the same as the analysis of XO ki.

*PCR Analyses for XO ki genotyping* – Genomic DNA was isolated from mouse-tail biopsy. Thirty-ng of template DNA was dissolved in 15 µl of 0.2 mM each dNTP with 5 units of PrimeSTAR HS DNA Polymerase (TaKaRa) and 1.2 µmol of each of the two primers in PrimerSTAR Buffer. Forward primer was derived from the short arm region in the targeting vector, 5'-CAGTGTCCTATCTCCTTTCTCGTTA-3', reverse primer from the long arm region in the targeting vector, 5'-TCCCAAATGAAACAAGGAATATAA-3'. The PCR was carried out for 37 cycles in a thermal cycle (Perkin-Elmer). Each cycle consisted of denaturation for 10 sec at 98°C, annealing for 5 sec at 55°C, and extension for 1 min at 72°C. A reaction sample was run in a 1% agarose gel by standard procedures.

*PCR Analyses for XDH ki genotyping* – Preparation of genomic DNA and PCR reaction mixture was same as the B6xo genotyping. Forward primer was derived from the long arm region in the targeting vector, 5'-CTGACACATGGAGGTACTGAGATG-3', reverse primer from the short arm region in the targeting vector, 5'-ATATCAATCCAGCACCATTAGCC-3'. The PCR was carried out for 35 cycles in a thermal cycle (Perkin-Elmer). Each cycle consisted of denaturation for 10 sec at 98°C, annealing for 5 sec at 60°C, and extension for 1 min at 72°C. A reaction sample was run in a 1% agarose gel by standard procedures.

# Supplementary Figure 1

A)

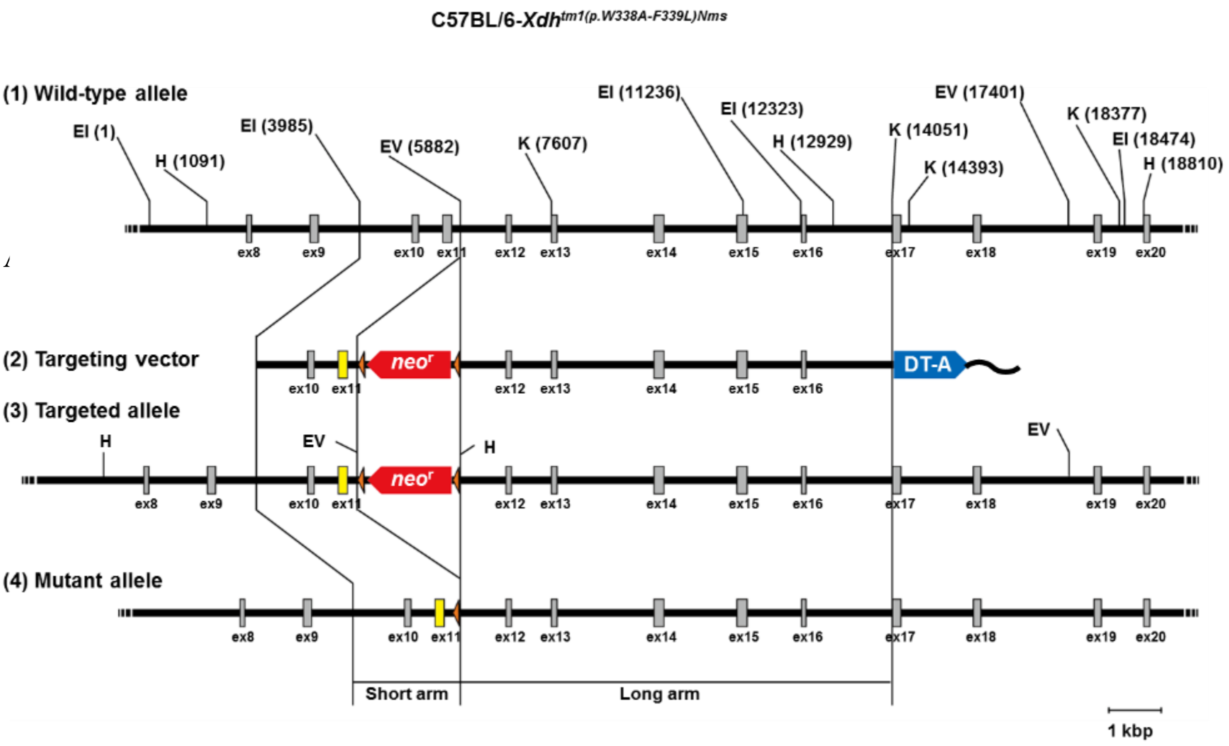


B)

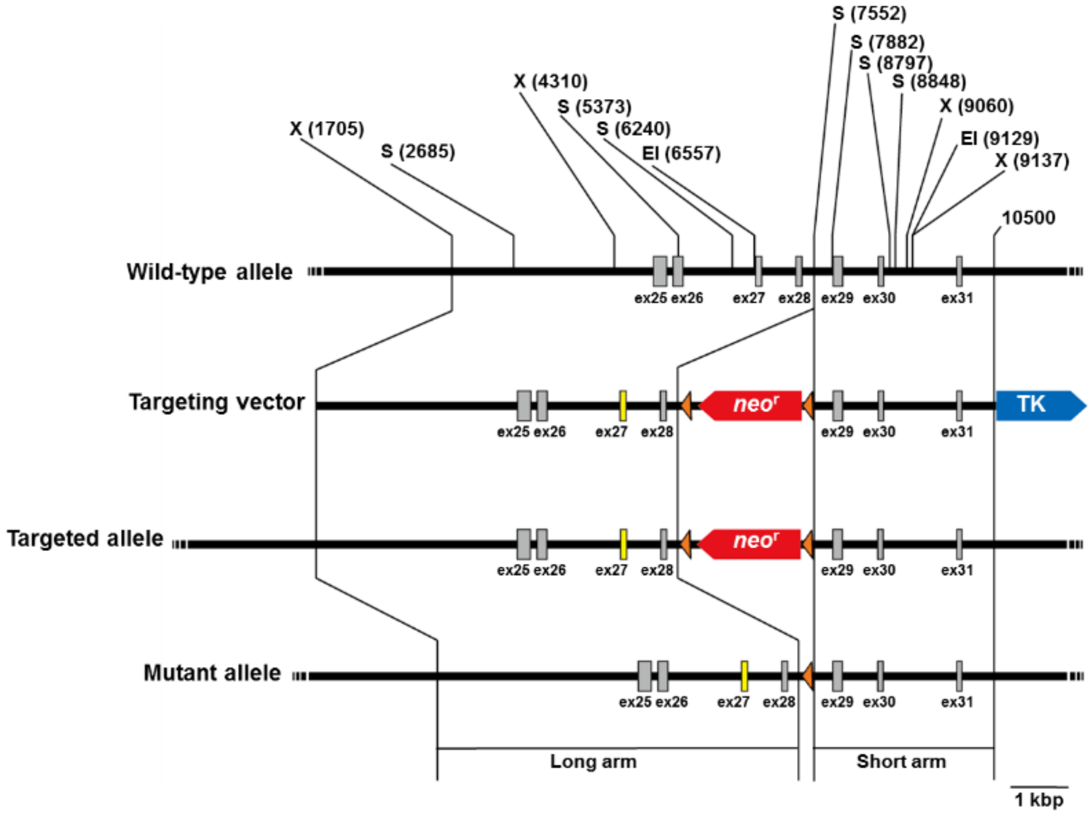

**Supplementary Figure 1: Strategy for targeted mutation of the murine Xdh gene**.

A, Targeted mutation of the gene encoding murine Xdh to XO-locked mutant. W338A/F339L mutation was introduced into exon 11. (1) Restriction endonuclease cleavage map and exon-intron organization of the wild-type Xdh gene. Restriction sites: EI, EcoR I; EV, EcoR V; H, Hind III; K, Kpn I. Filled boxes indicated the exons and those regions indicate the coding region of the murine Xdh gene. (2) Targeting vector used to insert the W338A/F339L mutation into the Xdh gene. Mutation site indicates yellow box. For the selection marker, a neomycin-resistance expression cassette (neor) was inserted at the EcoR V site posteriorly-located exon 11. A diphtheriae toxin A subunit (DTA) expression cassette added to the 3' end was used to eliminate clones having random integration of the targeting vector. (3) Restriction map and genomic structure of mutated Xdh gene after homologus recombination. (4) The gene structure after removal of neor by Cre recombinase treatment. Orange triangle indicate loxP site. The nucleotide sequence is numbered from the 5′ end of the cloned Xdh fragment and put the number in the parentheses of restriction enzyme site. B) Targeted mutation of the gene encoding murine Xdh to XDH-stable mutant. C995R mutation was introduced into exon 27. (1) Restriction endonuclease cleavage map and exon-intron organization of the wild-type Xdh gene. Restriction sites: EI, EcoR I; S, Sac I; X, Xba I. Filled boxes indicated the exons and those regions indicate the coding region of the murine Xdh gene. (2) Targeting vector used to insert the C995R mutation into the Xdh gene. Mutation site indicates yellow box. For the selection marker, a neomycin-resistance expression cassette (neor) was inserted at the EcoR V site posteriorly-located exon 28. A herpes simplex virus type 1 thymidine kinase (TK) expression cassette added to the 3' end was used to eliminate clones having random integration of the targeting vector. (3) Restriction map and genomic structure of mutated Xdh gene after homologus recombination. (4) The gene structure after removal of neor by Cre recombinase treatment. Orange triangle indicate loxP site. The nucleotide sequence is numbered from the 5′

end of the cloned Xdh fragment and put the number in the parentheses of restriction enzyme site.

## Supplementary Figure 2

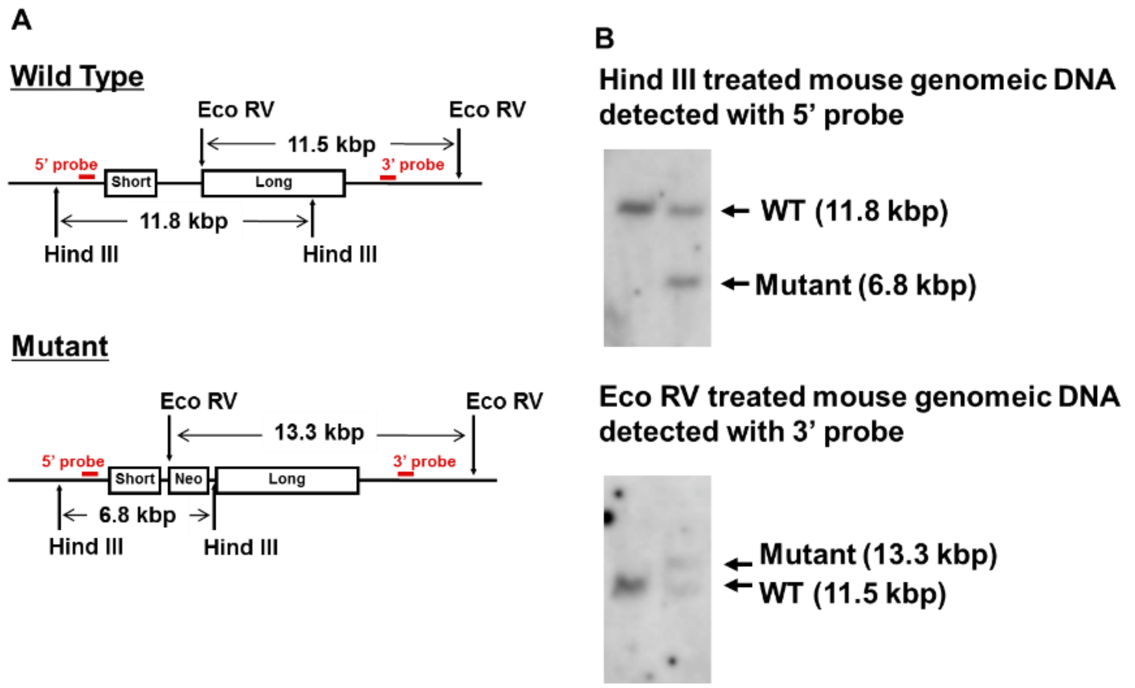


**Supplementary Figure 2: Southern blot analysis of the XO ki-Neor mouse genomic DNA**

A) Gene structure and restriction endonuclease map near the mutation site. The regions indicated as 5'-probe and 3'-probe were used to screen for homologus recombinants. Expected DNA fragments of wild-type and mutant alleles after digestion of genomic DNA with Hind III and EcoR V were hybridized with probes 5' probe and 3' probe, respectively. B) Southern blot analysis of genomic DNA from wild-type and F1 hetero mouse with Hind III (upper) or EcoR V (lower). The sizes of hybridizing DNA fragments are given on the right. lane 1; C57BL/6 (Wild type), lane 2; F1 hetero-mouse of XOR gene-modified C57BL/6 (W338A/F339L mutant).

**Supplementary Figure 3**

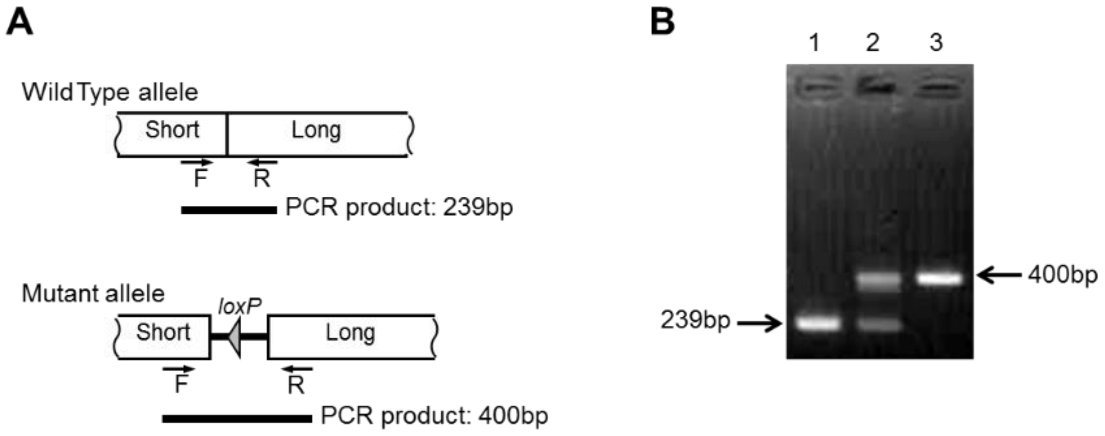


**Supplementary Figure 3: PCR identification of W338A/F339L homologous recombinants**.
A) Primer designation for PCR to confirm wild type and the XO-locked knock-in (Mutant) allele and the predicted size of PCR products. B) Representative gel image for wild-type (lane 1), B6xo heterozygote (lane 2), and B6xo homozygote (lane 3).

## Supplementary Figure 4

A)

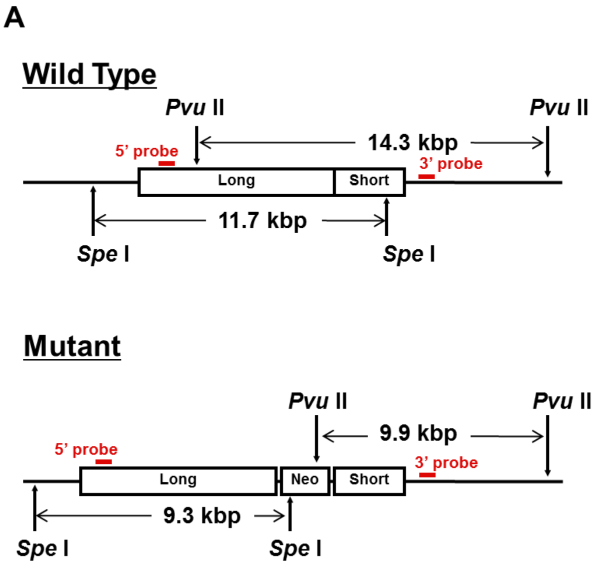


B)

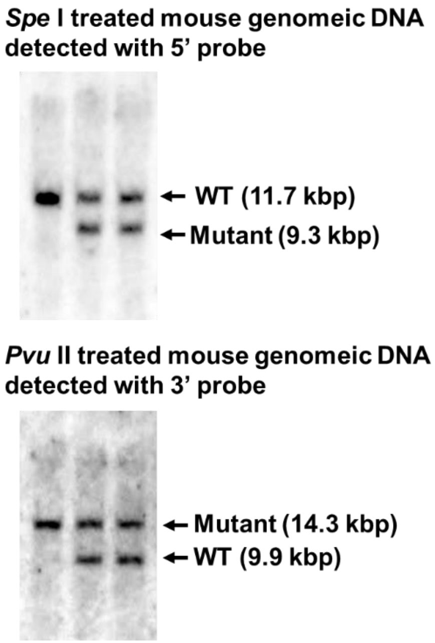

**Supplementary Figure 4: Southern blot analysis of the XDH ki-Neor mouse genomic DNA**.

A) Gene structure and restriction endonuclease map near the mutation site. The regions indicated as 5‘-probe and 3’probe were used to screen for homologus recombinants. Expected DNA fragments of wild-type and mutant alleles after digestion of genomic DNA with Spe I and Pvu II were hybridized with probes 5’ probe and 3’ probe, respectively. B) Southern blot analysis of genomic DNA from wild-type and F1 hetero mouse with Spe I (upper) or Pvu II (lower). The sizes of hybridizing DNA fragments are given on the right. lane 1; C57BL/6 (Wild type), lane 2 and 3; F1 heteromice of XOR gene-modified C57BL/6 (W338A/F339L mutant).

## Supplementary Figure 5

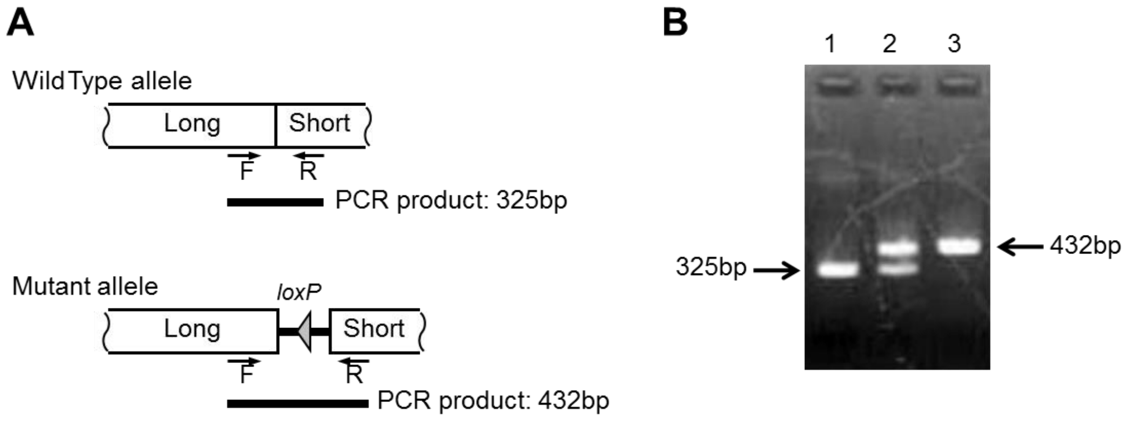


**Supplementary Figure 5: PCR identification of C995R homologous recombinants**
A) Primer designation for PCR to confirm wild type and the XDH-locked knock-in (Mutant) allele and the predicted size of PCR products. B) Representative gel image for wild-type (lane 1), B6 XDH heterozygote (lane 2), and B6 XDH homozygote (lane 3).

**Supplementary Figure 6**

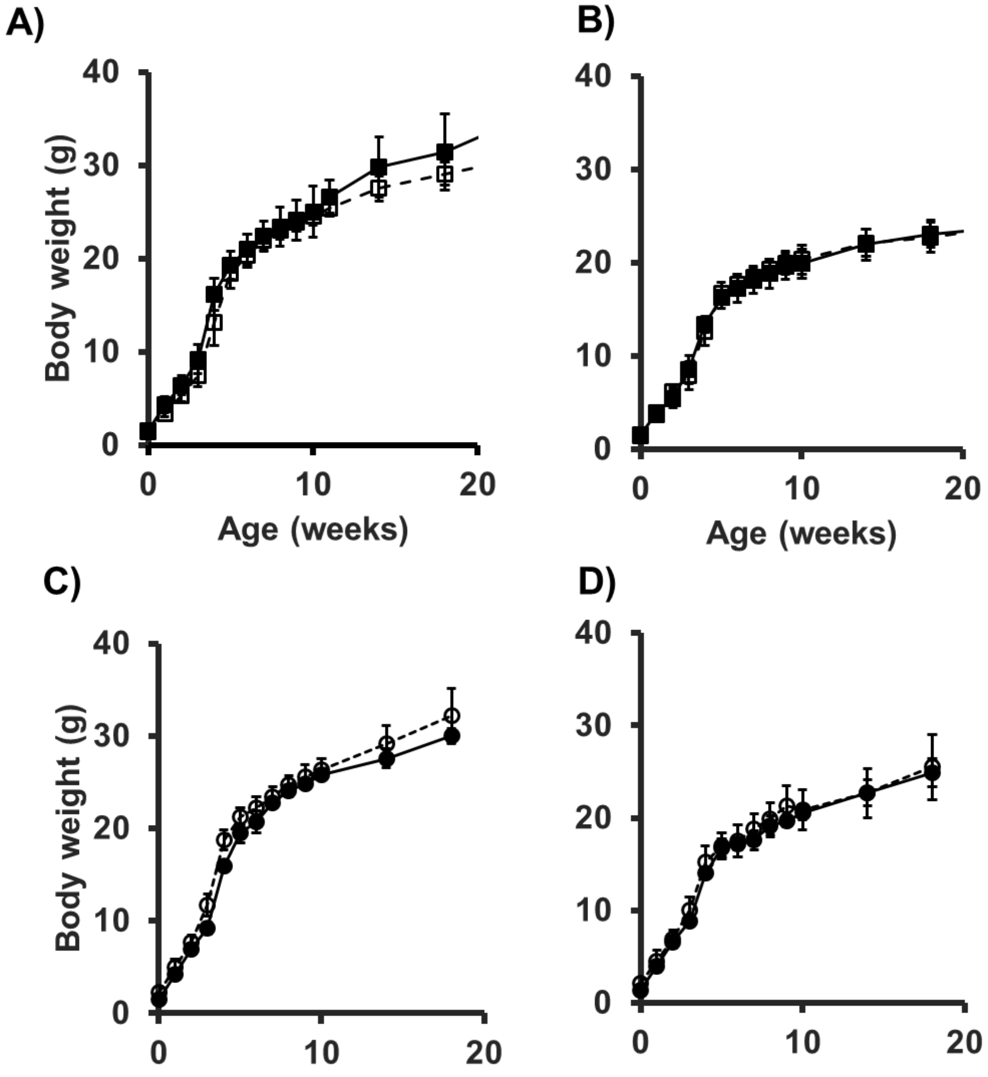


**Supplementary Figure 6: Growth was evaluated by body weight gain for up to 19 weeks in male (A and C) and female (B and D) XDH ki (A, B) and XO ki (C, D) mice.** There was no difference between the growth of XOR mutant mice (black line) and that of wild type mice (dotted line). Results are the mean +/- SD. The numbers of mice evaluated for each genotype are as follows; wild type littermate for XO ki (male n = 8, female n = 15), XO ki mice (male n = 10, female n = 9), littermate for XDH ki (male n = 9, female n = 13) and XDH ki mice (male n = 7, female n = 10).

**Supplementary Figure 7**

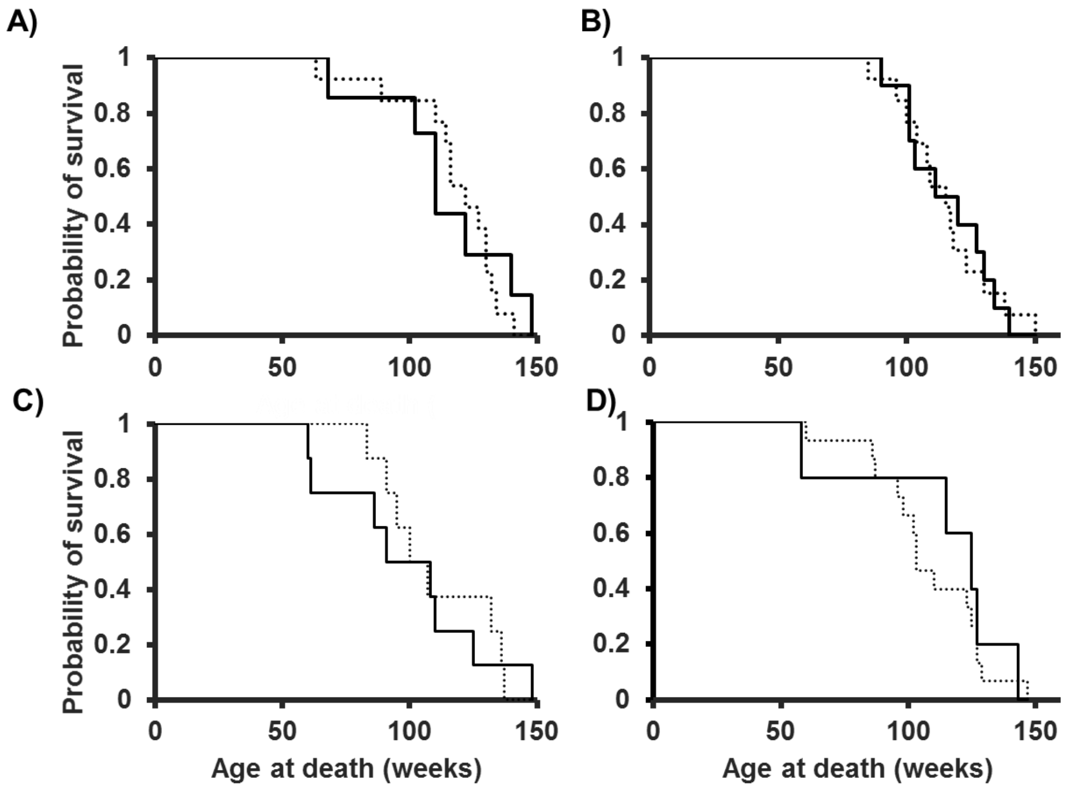


**Supplementary Figure 7: The estimation of survival distribution for XDH ki (A: male, B: female) and XO ki (C: male, D: female) mice over 150 weeks compared with WT mice by the Kaplan-Meier method.** There was no noticeable difference in survival between XOR mutant mice (black line) and WT mice (dotted line). The numbers of mice evaluated for each genotype are as follows; wild type littermate for XO ki (male n = 8, female n = 13), XO ki mice (male n = 8, female n = 5), littermate for XDH ki (male n = 13, female n = 13) and XDH ki mice (male n = 7, female n = 10).

**Supplementary Figure 8**

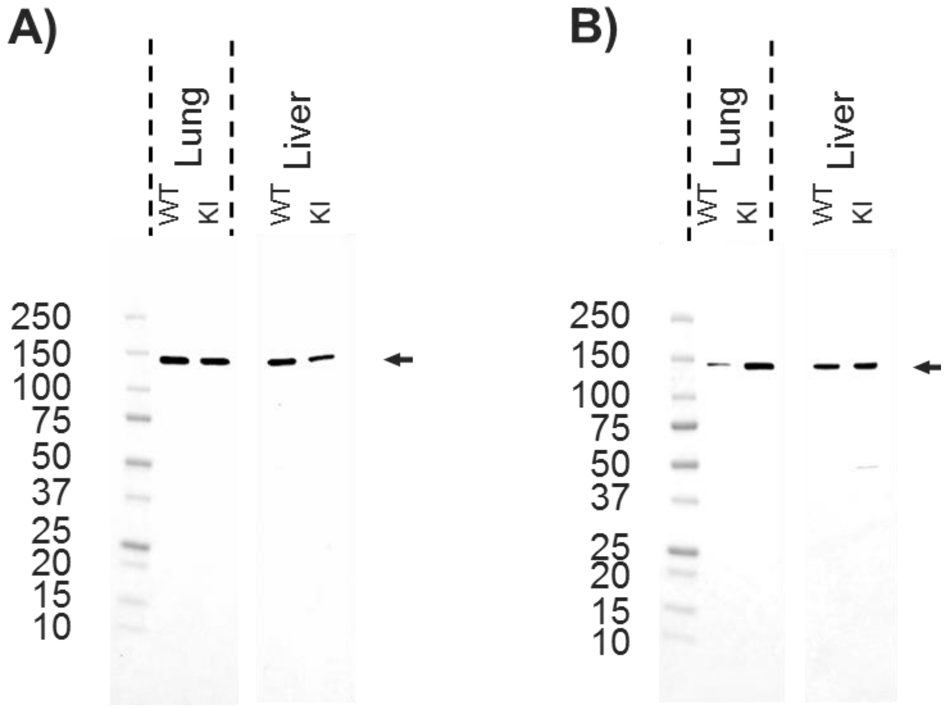


**Supplementary Figure 8: Western blot analysis of lung and liver proteins from XDH ki mice (A) or XO ki mice (B).** An anti-rat XOR antibody, which cross-reacts with purified mutated XOR proteins, revealed a 150 kD band in both XO ki and XDH ki lung and liver homogenates similar in size and intensity to that in wild-type counterparts.

**Supplementary Figure 9**

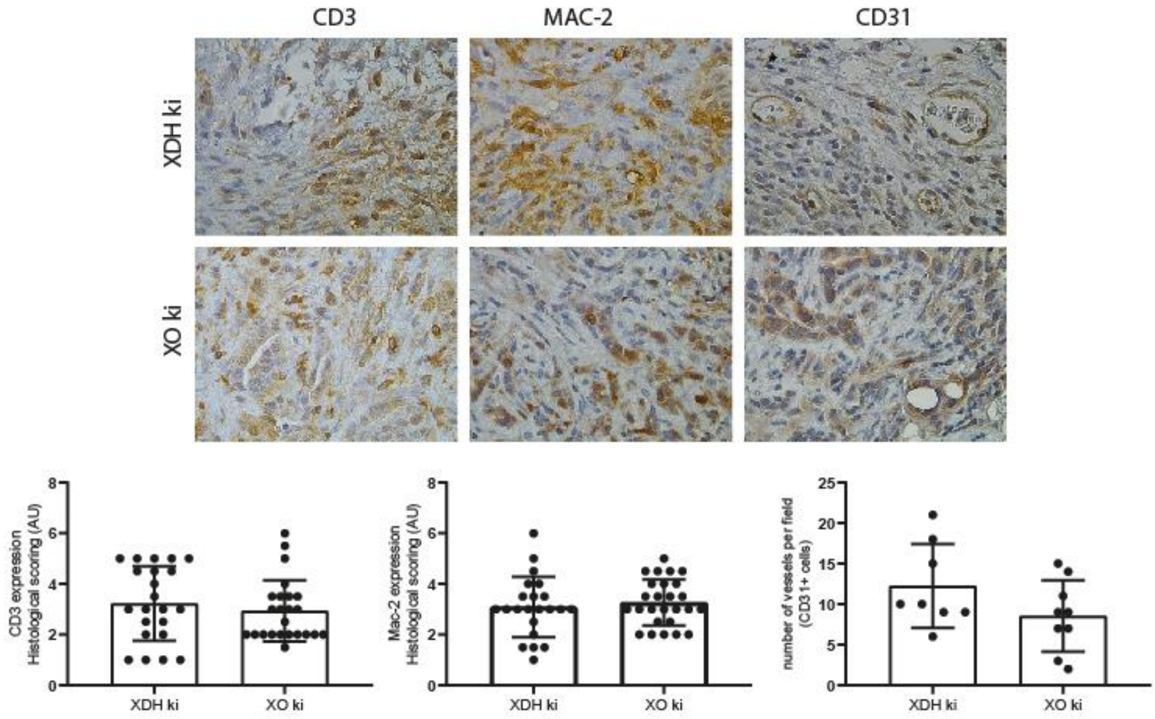


**Supplementary Figure 9:** A) CMT93 tumor cells were transferred subcutaneously in the flank of XDH ki and XO ki mice. Tumors were resected 15 days after inoculation and subjected to immunohistochemistry for T cells (anti CD3), macrophages (anti MAC-2) and endothelial cells (anti CD31) (X400 magnification). B) Corresponding immunohistological scoring (at least 3 mice per group with at least 3 different fields analyzed). No significant differences were found.
